# Natural variations in *TT8* and its neighboring *STK* confer yellow seed with elevated oil content in *Brassica juncea*

**DOI:** 10.1073/pnas.2417264122

**Published:** 2025-01-30

**Authors:** Lunwen Qian, Liu Yang, Xianjun Liu, Tianyi Wang, Lei Kang, Hao Chen, Yin Lu, Yukun Zhang, Shujie Yang, Liang You, Min Yao, Xingru Xiang, Kan Cui, Ying Guo, Bin Yang, Mingli Yan, Shitou Xia, Jinling Meng, Tao Lin, Annaliese S. Mason, Rod J. Snowdon, Zhongsong Liu

**Affiliations:** ^a^College of Agronomy, Hunan Agricultural University, Changsha 410128, China; ^b^Molecular Rapeseed Breeding Team, Yuelushan Laboratory, Changsha 410128, China; ^c^Smartgenomics Technology Institute, Tianjin 301700, China; ^d^Hunan Provincial Key Laboratory of Phytohormones and Growth Development, College of Bioscience and Biotechnology, Hunan Agricultural University, Changsha 410128, China; ^e^National Key Laboratory of Crop Genetic Improvement, Key Laboratory of Rapeseed Genetic Improvement, Ministry of Agriculture China, Huazhong Agricultural University, Wuhan 430072, China; ^f^College of Horticulture, China Agricultural University, Beijing 100193, China; ^g^Plant Breeding Department, The University of Bonn, Bonn 53115, Germany; ^h^Department of Plant Breeding, Justus Liebig University Giessen, Giessen 35392, Germany

**Keywords:** oil content, STK, TT8, co-evolution, haplotype

## Abstract

Seed color is a critical quality trait in numerous plant species. In oilseed *Brassica* crops, including rapeseed and mustard, yellow seeds are distinguished by their significantly higher oil content and faster germination rates compared to black or brown counterparts. Despite the agronomic significance of the yellow seeds being a prime breeding target, the mechanisms underlying elevated oil content remain obscure. In this study, we assembled the first telomere-to-telomere (T2T) genome of *B. juncea* and further investigated the genetic regulation, molecular mechanism, and the evolutionary history of yellow seeds in *B. juncea.* Through an analysis of allelic variation in the *TRANSPARENT TESTA 8* (*TT8*) genes across 1,002 worldwide *B*. *juncea* accessions, we traced the single origin of yellow seeds to approximately 2,300 y ago in Southwestern China. Furthermore, we discovered the MADS-box gene *SEEDSTICK* (*STK*) coevolved with *TT8*, and they coordinately regulated seed size, oil accumulation, and seed coat proportion in *B. juncea*. These findings open broad avenues for targeted breeding of yellow-seeded *Brassica* crops with elevated oil content.

## Significance

The yellow seed trait is associated with higher oil content and quicker seed germination in *Brassica juncea* (L.) Czern & Coss. However, the origin and evolution of yellow seed, an important agronomical trait, remains to be elucidated. In this study, we assembled the first telomere-to-telomere (T2T) genome of *B. juncea* and proved that the natural yellow seed is produced by *TRANSPARENT TESTA 8* (*TT8*) variation in *B. juncea*. Geographic distribution and haplotype network analysis of *TT8s* in 1,002 accessions revealed a single origin of yellow seeds in Southwestern China. We found antagonistic *TT8* and *SEEDSTICK* (*STK*) form a transcriptional regulatory network that regulates higher seed oil accumulation and bigger seed size during the evolution of yellow-seeded *B. juncea*.

Seeds are propagules for flowering plants and vital agricultural products for many crop species. Seed color, a morphological trait exhibiting a wide range of variation, is frequently associated with seed germination and quality (1). In *Brassica* species, seeds often manifest as black or brown, while naturally occurring yellow-seeded variants have been found in *B. rapa*, *B. juncea*, and *B. carinata* (2). Since the 1960s, breeding programs have prioritized the selection of yellow seeds in oilseed *Brassica* due to their significantly higher oil content compared to black or brown counterparts (3). Genetically, the yellow seed trait in *B. juncea* is governed by duplicate recessive loci (4, 5). They have been mapped to the chromosomes A09 and B08 (6, 7), respectively, encoding the same bHLH transcription factor gene *TRANSPARENT TESTA 8* (*TT8*) (8, 9). *TT8* is one of some 20 known genes regulating flavonoid biosynthesis and seed color in *Arabidopsis* (10, 11). Our research revealed that the black- or brown-colored *Brassica* seeds deposit proanthocyanidins (PAs) in their testa, while the yellow-colored seed lacks PAs, rendering them transparent (12). However, the molecular mechanism underlying the regulation of seed color formation by *TT8* is not well understood in *B. juncea*.

Multiomics analysis showed that a regulatory hotspot on chromosome A09 carried *TT8* controlling seed coat percentage (SCP), seed oil content (SOC), and seed coat lignin content in *B. napus* (13, 14). RNA-seq analysis revealed that *TT8*, *ANTHOCYANIDIN REDUCTASE* (*ANR*), *WRINKLED 1* (*WRI1*), *TSO1*, *CYCLIN B 1;2* (*CYCB1*;*2*), etc., form a transcriptional regulatory network that control seed size, seed color, and oil content in *B. rapa* (15). Despite these findings, the molecular mechanism underlying the formation of yellow seeds and the reason for their high oil content remains obscure in *Brassica* species.

The renowned Russian geneticist Nicolai Vavilov discovered spontaneous yellow-seeded *B. juncea* mutants during an expedition to Xinjiang, China, in 1929 (16). The yellow-seeded accessions occurred exclusively in the Chinese and Eastern European *B. juncea* group, referred to as Oriental mustard, to distinguish these from the brown (or Indian) mustard from India and Pakistan (17). The yellow-seeded *B. juncea* accessions have been amassed in China (18) and exploited as critical germplasm in *Brassica* breeding endeavors (19). To date, little is known about the precise origin and timing of the spontaneous emergence of yellow-seeded *B. juncea* mutants. Tracing their origin is crucial for the collection, conservation, and targeted breeding of *Brassica* crops featuring yellow seed color.

In this study, we assembled the first *B. juncea* telomere-to-telomere (T2T) genome using the black-seeded parent of our mapping populations. We performed multiomics analyses to identify *TT8* genes and their central roles in controlling yellow seed color in *B. juncea*. In addition, by analyzing the allelic variations and haplotypes in 1,002 accessions worldwide, we revealed the origin of the yellow-seeded *B. juncea* in Southwestern China. Finally, using linkage disequilibrium (LD) analysis, we discovered that *TT8s* coevolved with their neighboring *SEEDSTICK* (*STK*) genes during the domestication of the yellow-seeded *B. juncea.* The genome-wide association study (GWAS), haplotype analysis, and transcriptome profiling showed that *TT8s* and *STKs* inhibited each other in gene expression and coregulated a couple of seed traits including oil content in *B. juncea*. These results paint a comprehensive picture of the genetic regulation, molecular mechanisms, and evolutionary history of the yellow seed color trait in *B. juncea* and will help establish an alternative paradigm in the genetic improvement of yellow-seeded *Brassica* crops with elevated oil content.

## Results

### A T2T Genome Assembly of *B. juncea*.

**Fig. 1.**
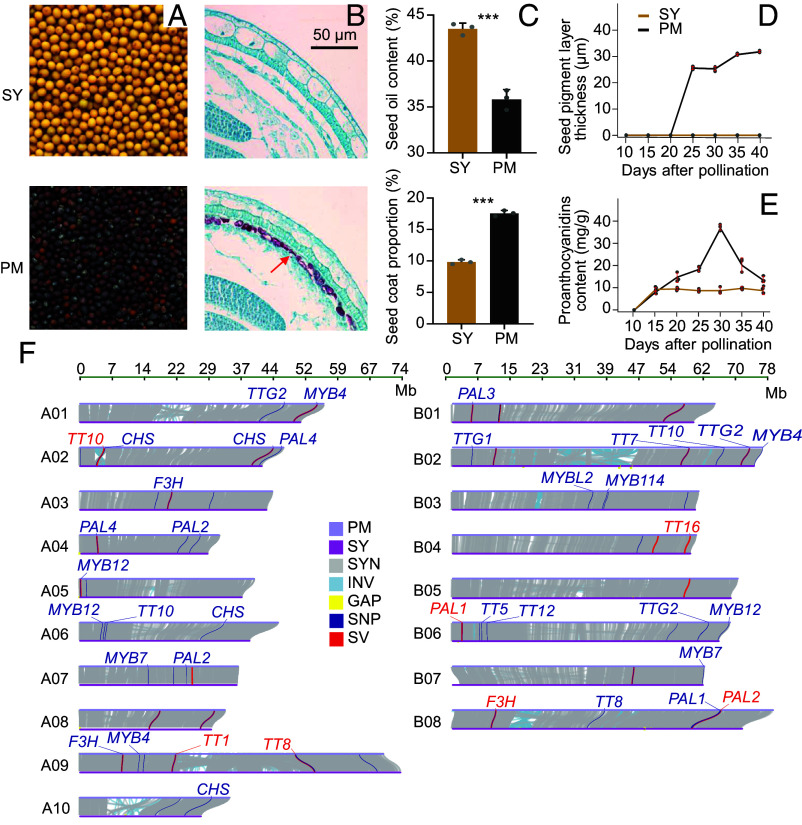
Phenotyping and structural variation analysis of flavonoid biosynthetic genes between *B. juncea* var. SY and Purple Leaf Mustard (PM) and the T2T PM genome assembly. (*A*) The photographs of mature seeds from SY and PM. (*B*) Microscopy of Safranin O and Fast Green‐ stained transverse sections from seeds of SY and PM 25 d after pollination (DAP). The arrowhead indicates the red-stained PAs deposited in the endothelial cells of the seed coats. (Scale bars, 50 µm.) (*C*) SY have higher SOC and less SCP than PM. (*D*) The pigment layer is thicker in PM than in SY from 20 DAP onward. (*E*) PM accumulates more PAs in seed coat than SY from 15 to 40 DAP. (*F*) Chromosomal features, variations, and synteny landscape of the *B. juncea* var. PM genome. Comparison in sequences of flavonoid biosynthetic genes between SY and PM. SYN, Collinear region; INV, Inversion region; GAP, Uncovered regions; SNP, Single-nucleotide polymorphisms; SV, Structural variation.

The *B. juncea* var. Sichuan Yellow (SY), a landrace from Sichuan, China, produces yellow seeds with no or less pigmented layer in their testa, low seed coat proportion (SCP) and PAs content, and high oil content. Contrary to SY, *B. juncea* var. Purple-leaf Mustard (PM), a landrace from Hunan, China, bears black seeds with a thick pigmented layer, higher SCP and PAs content, and lower oil content (Fig. 1 *A*–*E*). To uncover the genomic and genetic basis for variations in these traits between these contrasting accessions, we assembled the genome of PM after whole-genome sequencing of SY (20).

For PM, we first used HiFIasm (21) for the backbone genome assembly using 87.65Gbp PacBio HiFi reads and 57.70 Gbp ultralong ONT reads, which generated an initial assembly of 142 contigs with an N50 length of 58.47 Mbp (Dataset S1). Second, we used NextDenovo (22) to assemble the ONT reads, HiCanu (23) and Verkko (24) to assemble the HiFi reads, and NextPolish (25) to polish the assembly. Twenty-two nonredundant contigs were anchored and oriented onto 18 pseudomolecules using the interaction map generated by 185.78Gbp Hi-C data (*SI Appendix*, Fig. S1), leaving only four gaps. Third, we filled these gaps using Ragtag (26) with the NextDenovo-, the HiCanu-, and the Verkko-based genomes and generated a gap-free PM genome assembly. Furthermore, we identified all telomeres using Tidk (https://github.com/tolkit/telomeric-identifier) and Minimap2 (27), and all contiguous centromeric regions (*SI Appendix*, Fig. S2 and Dataset S2). The final PM genome assembly is 981.82 Mbp long (Datasets S3 and S4). To our knowledge, the PM genome is the first T2T assembly of *B. juncea*, which covered over 99.9% of HiFi reads and identified 99.7% of genes in the BUSCO (28) dataset (Dataset S5). For the PM genome, about 55.33% was annotated as repetitive elements (Dataset S6) and a total of 84,664 protein-coding genes were predicted with 96.6% (81,803) being functionally annotated (Datasets S7 and S8). Its LTR assembly index(29) reached 16.64 (Dataset S5), indicating high assembly integrity even in repeat-rich regions. Taken together, this PM genome assembly is superior to previous *B. juncea* ones (20, 30, 31), which provides a better reference genome for genomics and gene identification.

Utilizing the T2T PM genome, we identified a total of 325 genes for flavonoid biosynthesis by homolog search (Dataset S9). We compared these genes in sequence between SY and PM and found 1,762 SNPs in 55 genes (Fig. 1*F* and Dataset S10), as well as 16 SVs (>50 bp) in eleven genes (Fig. 1*F* and Dataset S11), which is the genome-wide identification and sequence comparison of flavonoid synthesis genes, laying the foundation for research on flavonoid accumulation and pigmentation of plant organs in *B. juncea*.

### Regulation of the Yellow Seed Trait by *TT8* Genes in *B. juncea*.

#### GWAS of seed color.

Using a panel of 480 *B. juncea* accessions (Dataset S12), we scanned genomic regions that showed marked reductions in nucleotide diversity by comparing black/brown- with yellow-seeded accessions. Seventeen putative selective sweeps were identified, where 17 genes were reported to be involved in flavonoid biosynthesis (*SI Appendix*, Fig. S3 and Dataset S13). Simultaneously, we detected a total of 27 candidate genes significantly associated with seed color by the GWAS with significant *p-*value thresholds (*P* < 10^−6^) (Dataset S14). Only *Arabidopsis* homologous *TT8* genes were detected by both methods.

**Fig. 2.**
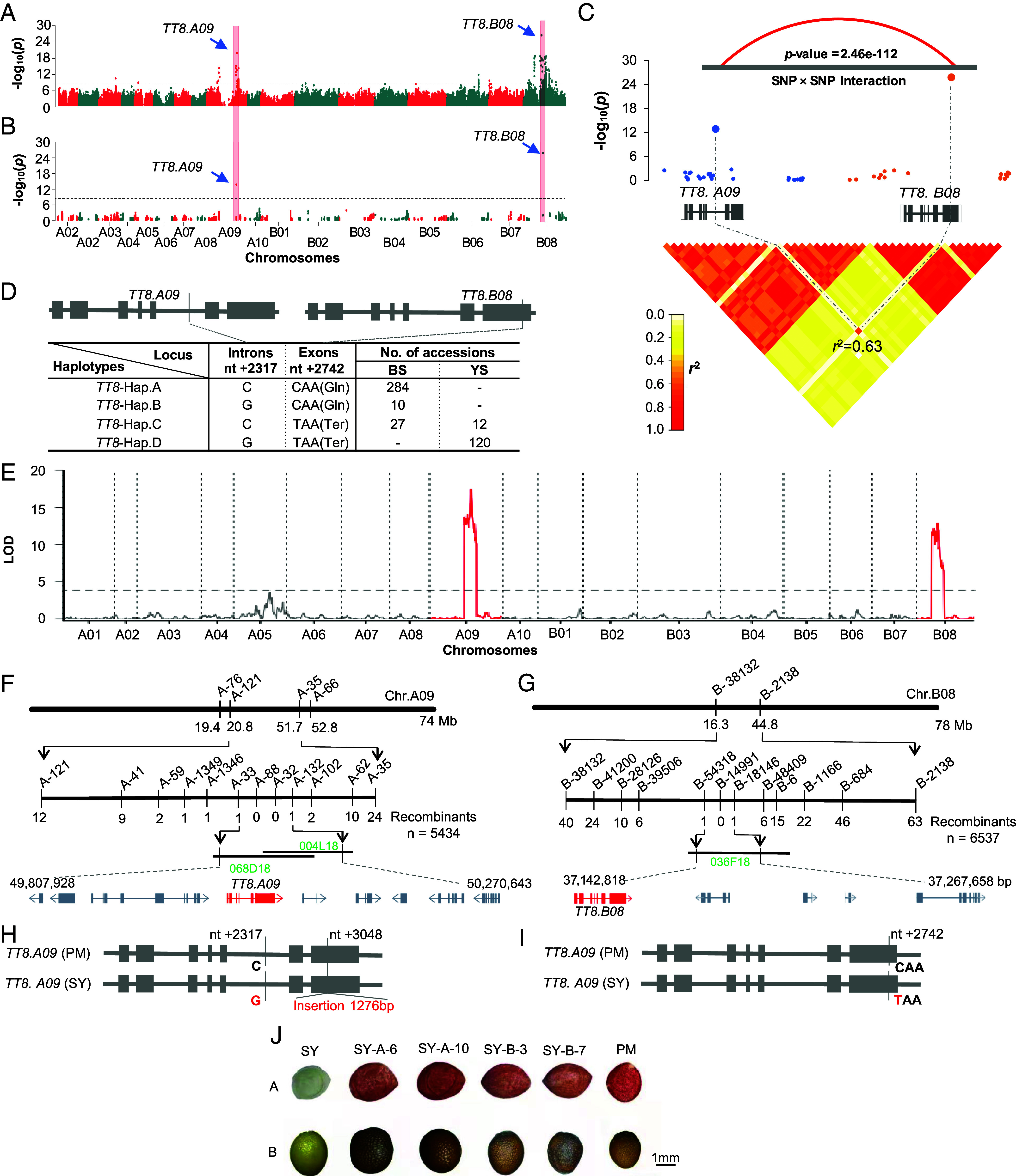
Mapping and cloning of the genes for seed color in *B. juncea*. Genome-wide association analysis (*A*) and candidate gene association analysis (*B*) detected *TRANSPARENT TESTA* (*TT8*) significantly associated with seed color on chromosomes A09 and B08. The gray dotted lines indicate the significance threshold (−log_10_(*P*)= 6.0). (*C*) One single nucleotide polymorphism (SNP) in intron 5 and one in exon 7 of *TT8*s from chromosomes A09 and B08, respectively, showed significant association with seed color. The red curve and heatmaps show a strong interaction and close linkage between these SNPs from *TT8s*, respectively. (*D*) Four haplotypes with frequency greater than 0.01 were identified in two *TT8* gene regions, which corresponded to accessions of different numbers and colors. (*E*) Two quantitative trait locus (QTLs) were identified for seed color in the recombinant inbred line (RIL) population from the cross of *B. juncea* var. SY with *B. juncea* var. Purple Leaf Mustard (PM). The y axis is the logarithm of the odds value. Fine-mapping for seed color in the A09 (*F*) and B08 (*G*) chromosome QTLs, respectively. (*H*) The 1,276-bp insertion in the seventh exon of *TT8*. *A09* in SY, and a base transversion from C of PM to G of SY at nucleotide 2,317. (*I*) A base transition *TT8*.*B08* from C of PM to T of SY at nucleotide 2,742. (*J*) Vanillin staining of seed coat and the phenotype of mature seeds of SY, SY transformants, and PM. A: Vanillin staining of seed coats 30 DAP; B: Phenotype of mature seeds; SY: Sichuan Yellow; SY-A-6, SY-A-10, SY-B-3, and SY-B-7: four positive transformants of SY with the *TT8.A09*-PM or *TT8.B08*-PM genes; PM: Purple-leaf Mustard. (Scale bar, 1 mm.)

As shown by the genome-wide scanning, the SNP (C→G, nt + 2,317, *P* = 1.28 × 10^−18^) in intron 5 of *TT8.A09* and the SNP (C→T, Gln→Ter, nt +2,742, *P* = 2.57 × 10^−25^) in exon 7 of *TT8. B08* showed the most significant association with the seed color, respectively (Fig. 2*A*). Using the flavonoid biosynthetic genes identified above, we did association analysis which showed these two SNPs were also significantly associated with seed color (Fig. 2*B*). These SNPs showed LD (*r*^2^ = 0.63) and strong interaction (*P*-value = 2.46e-112, Fig. 2*C*), and the *TT8*-Hap.D (G + T) in 148 accessions, all with yellow seeds (Fig. 2*D*). These results unambiguously show that the *TT8* genes are responsible for the yellow seed trait in natural *B. juncea* accessions.

#### Mapping and cloning of TT8s.

To confirm the above results, we used a population of 172 F_6_ RIL (RILs; *SI Appendix*, Fig. S4) from crossing SY with PM to map the genes for seed color. As illustrated in Fig. 2*E*, the seed color loci were located on chromosomes A09 (20,811,121 to 51,710,866 Mb) and B08 (16,287,745 to 44,478,602 Mb), respectively, confirming previous results (4–7, 32, 33). Furthermore, we constructed two large BC_8_F_2_ mapping populations segregating only at a single seed color locus (Fig. 2 *F* and *G* and *SI Appendix*, Fig. S4). Genotyping of recessive yellow-seeded individuals (Dataset S15) mapped the seed color loci between the markers A33 and A132, and B28126 and B54318(Fig. 2 *F* and *G* and Dataset S16). These flanking markers were used to screen a BAC library constructed from PM. The positive BAC clones were sequenced and annotated, predicting the genes BjuA09G32400PM and BjuB08G17740PM, which are homologous to *TT8* in *Arabidopsis* (*TT8.A09* and *TT8.B08*) (Dataset S17). The *TT8.A09* allele from SY was 4,827 bp long, 1,276 bp longer than that from PM due to an insertion in exon 7 between nt 3,048 and 3,049, and contained a base transversion(C/G) in intron 5 for nt 2,317 (Fig. 2*H*), while a base transition (C/T) in *TT8.B08* was found in exon 7 for nt 2,742 (Fig. 2*I*).

The genes *TT8.A09* and *TT8.B08* from PM were transformed separately into SY, resulting in ten *TT8.A09* and five *TT8.B08* positive transformants verified by PCR amplification. Phenotyping seed color and staining of seed coats by vanillin solution (12) showed all transformants produced black seeds with their testa stained red (Fig. 2*J*), supporting that the *TT8* controls seed color in *B. juncea*.

### Regulatory Mechanism of Seed Color by *TT8s* in *B. juncea*.

**Fig. 3.**
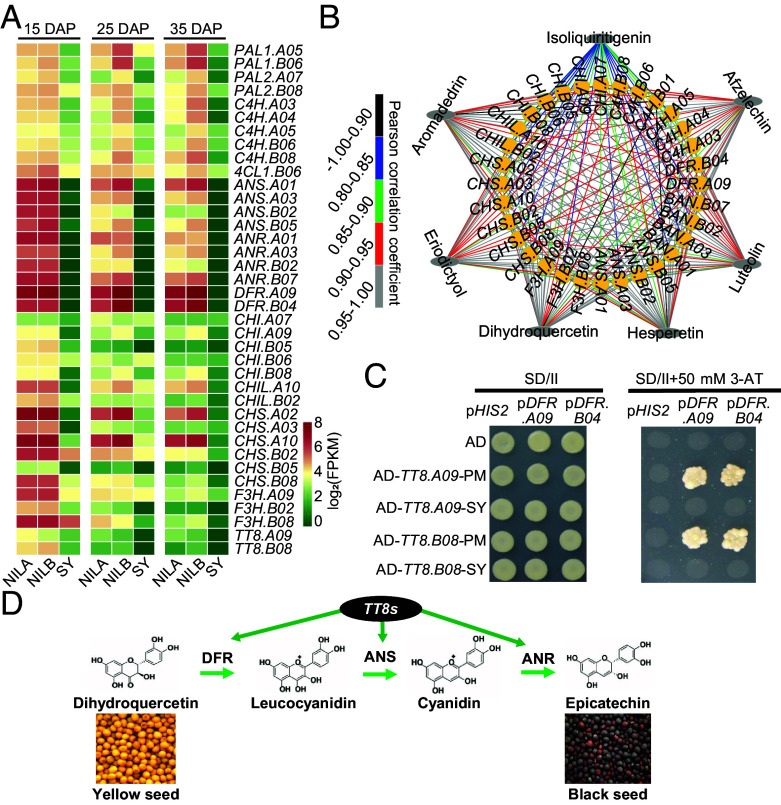
The molecular mechanism of yellow seed formation in *B. juncea*. (*A*) Expression analysis of flavonoid biosynthetic genes in seed coat 15, 25, and 35 DAP among *B. juncea* var. SY and its NILs A and B (NILA and NILB). (*B*) Connection network between the structural genes and flavonoid metabolites. Yellow circles represent the genes, while the gray oval boxes represent metabolites. (*C*) Interaction of *TRANSPARENT TESTAS* (*TT8s*) with the promoters of *DIHYDROFLAVONOL 4-REDUCTASES* (*DFRs*) in yeast one-hybrid assays. The different plasmid combinations cotransformed into yeast cells. Survival of the cells in Synthetic defined (SD) medium without histidine and adenine (SD + 50 mM 3-AT) and milk white colonies indicated an interaction between the two cotransformed gene products. The empty prey vector (AD) was used as a negative control. (*D*) Regulatory mechanism of yellow seed color by *TT8* mutation in *B. juncea*.

To understand how *TT8* genes regulate seed color in *B. juncea*, transcriptome profiling was performed to compare gene expression in seed coats of SY and its brown-seeded near-isogenic lines (NILs) at 15, 25, and 35 DAP (days after pollination; *SI Appendix*, Fig. S5). Among 96,332 de novo assembled unigenes, 27 genes were up-regulated while 97 down-regulated (*SI Appendix*, Fig. S5 and Dataset S18). KEGG analysis revealed that twelve down-regulated genes are involved in the flavonoid biosynthesis (Fig. 3*A* and Dataset S18). Notably, the expression of *TT8*, *DIHYDROFLAVONOL 4-REDUCTASE* (*DFR*), *ANTHOCYANIDIN SYNTHASE* (*ANS*), and *ANR* is absent in the testa of SY, all of which are essential for PA biosynthesis.

Indeed, we detected 143 flavonoids in the testa of mature seeds of SY and its NILs using quantitative UPLC-ESI-MS/MS (Dataset S19). Of these flavonoids, 37 were different in content, with PAs and their precursors epicatechin completely absent from SY testa (*SI Appendix*, Fig. S6 and Dataset S20). The content of seven flavonoid metabolites correlated significantly with the expression level of the genes *DFR*, *ANS,* and *ANR* (*r* > 0.80) (Fig. 3*B*, *SI Appendix*, Fig. S7, and Dataset S21), supporting that PAs are responsible for seed coat pigmentation in *Brassica* species (34–37).

Previous studies demonstrated that TT8, a bHLH transcription factor, drives the expression of *DFRs*, thereby activating the biosynthesis of flava-3-ols and subsequently PAs in *Arabidopsis* (38, 39). Utilizing a yeast one-hybrid assay, we found that the mutated *tt8s* from SY failed to bind to the *DFR* promotor, whereas *TT8* from PM remained functional (Fig. 3*C*). Collectively, these results reveal that *B. juncea TT8*s regulate seed color via the control of PA biosynthesis (Fig. 3*D*).

### Origin of the Yellow-Seeded *B. juncea* in Southwestern China.

*B. juncea* is distributed globally, and our studies traced its single origin to West Asia, approximately 8,000 to 14,000 y ago (20). However, the exact geographical origin of yellow-seeded *B. juncea* remains undetermined. To address this issue, we characterized the allelic variation of *TT8*.*A09* and *TT8*.*B08* (hereafter referred to as *TT8.A* and *TT8*.*B*) in 1,002 *B. juncea* accessions collected from around the world (Dataset S22). Seven *TT8.A* and six *TT8.B* alleles were identified by PCR amplification, respectively (*SI Appendix*, Fig. S8 *A* and *C*). The wild-type alleles *TT8.A* and *TT8.B*, identical to those of PM, were identified in 616 and 594 black-seeded accessions, respectively. The alleles *tt8.a1* and *tt8.b1,* identical to those from SY, the major mutant type, were found in 49 black- and 314 yellow-seeded accessions, and in 74 black- and 306 yellow-seeded ones, respectively. The minor or rare alleles *tt8.a2*, *tt8.a3, tt8.a6,* and *tt8.b2* were detected only in the yellow-seeded accessions, while *tt8.a5* and *tt8.b3*, *tt8.b4,* and *tt8.b5* only in the black-seeded accessions, although *tt8.a4* was found in both yellow- and black-seeded accessions. Complementation tests showed all mutated *tt8* alleles lost their function, leading to production of yellow seeds (Dataset S23).

We constructed a phylogenetic tree based on the concordance of point mutations and inferred the *tt8.a* alleles formed 2,800 to 1,000 y ago, while the *tt8.b* alleles did 3,500 to 2,000 y ago, with the most common *tt8.a1* and *tt8.b1* being the earliest mutations to occur (*SI Appendix*, Fig. S8 *B* and *D*).

**Fig. 4.**
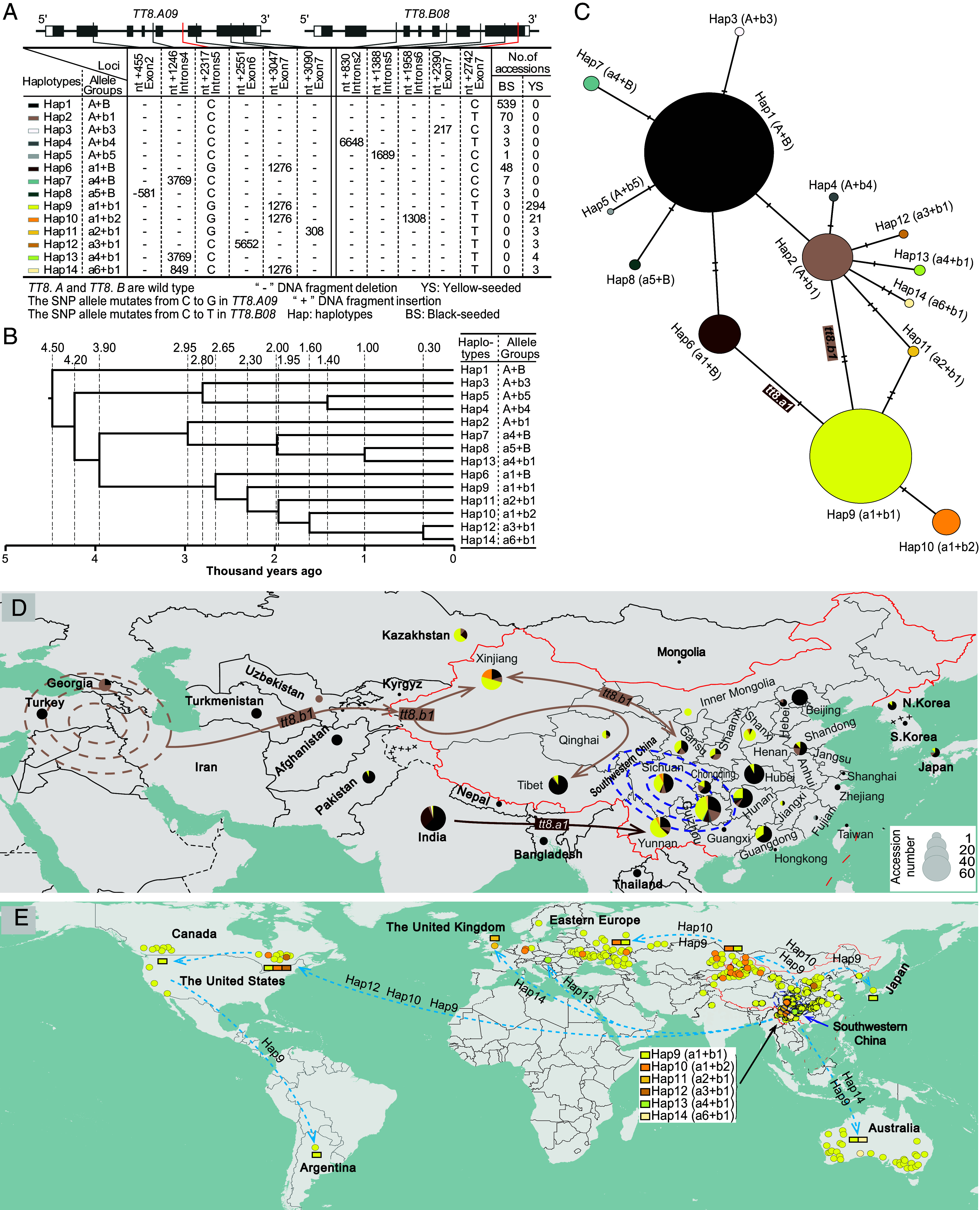
Origin and spread of the yellow seed trait in *B. juncea.* (*A*) Allelic variation in *TT8* genes. A total of 14 haplotypes combining *TT8.A09* and *TT8.B08* were detected, which corresponded to the seed color phenotype in *B. juncea*. (*B*) The inferred phylogeny of mutation in *TT8* genes, shown with estimated divergence times based on the speciation time 8,000 to 14,000 y of *B. juncea* as the standard (20). (*C*) Haplotype network of the *TT8* genes. The network was constructed by utilizing mutated loci of the *TT8* gene regions in 1,002 *B. juncea* accessions. Each dot represents a haplotype, connected by lines in which each mark indicates a mutational step between connected haplotypes. The pie chart size is proportional to the number of accessions. (*D*) Country- and China’s province/region-specific distribution of accessions with different seed colors. (*E*) The routes by which the yellow seed variants of *B. juncea* spread.

Allelic variation of *TT8s* constituted 14 haplotypes (Haps) across the *B. juncea* accessions. Hap 1 is the wild type and Haps 2 to 8 had mutations at a single *TT8* gene, all of which were found in black/brown-seeded accessions, whereas Haps 9 to 14 with concurrent mutations at both *TT8*s were identified in yellow-seeded accessions (Fig. 4*A* and Dataset S22). Hap 9, the most prevalent yellow-seeded group, was found in 294 yellow-seeded accessions, whereas other yellow-seeded haplotypes occurred in dozens, even a few accessions (Fig. 4*A* and Dataset S22). Estimation of the divergence time of the yellow-seeded haplotypes indicated that Hap 9 originated earliest approximately 2,300 y ago, while Hap 12 and Hap 14 the most recently 300 y ago (Fig. 4*B*).

Haplotype network analysis showed that black/brown-seeded Hap 2 and Hap 6 served as critical bridges to the yellow-seeded Hap 9 (Fig. 4*C*). Hap 2 carrying the *tt8.a1* allele was mutated in India, then spread to Southwestern China (Fig. 4*D*) along the South Silk Road. In parallel, Hap 6 carrying *tt8.b1* was mutated in West Asia, the place of origin of *B. juncea* (20), and was then introduced to Northwestern China along the Steppe Route (Fig. 4*D*). These two haplotypes met in Southwestern China and evolved Hap 9 from introgressive hybridization (Fig. 4 *C* and *D*), which is consistent with our previous analyses of population structure (20) and organelle genomes (40) as well as the historical written records suggesting China, but not India (41), as the origin of yellow-seeded *B. juncea*. It is only in Southwestern China that all other yellow-seeded haplotypes were detected (Fig. 4*D*), supporting a single geographic origin of the yellow seed trait in Southwestern China.

In order to investigate the spread of yellow seeds across the globe, we calculated the geographic distribution of the haplotypes in all 328 yellow-seeded accessions (Fig. 4*E* and Dataset S24). Hap 9 was distributed over 17 countries, with China (172/328) and the former USSR (57/328) being major distribution areas, whereas Haps 10 to 14 were found only in two to five countries each, in each case including China (Fig. 4*E* and Dataset S24). Chinese mustard was introduced to the former USSR in the late 18th century (42) and France around 1860 (43). The United States and Canada received yellow-seeded mustard in the 1940s from China and Russia (44–46), while Australia's yellow-seeded mustard was introduced from the 1970s onward directly from China or via the United Kingdom (19). Yellow seededness is the desired target trait for breeding in oilseed and condiment *Brassica* crops. As listed in Dataset S24, Hap 9 (*tt8.a1*+*tt8.b1*) is the most prevalent among the bred yellow-seeded oilseed *B. juncea* varieties. Hap 10 (*tt8.a1*+*tt8.b2*) is utilized in Canadian (e.g., CBJ004) and Ukrainian (e.g., Ekla) *B. juncea* breeding programs when Zem-2 from China is used as the donor of zero erucic acid genes (47), while Hap 14 (*tt8.a6*+*tt8.b1*) is used for development of Australian early-maturing *B. juncea* lines, for example, JO009 (48). In summary, the yellow-seeded *B. juncea* originates from Southwestern China and was subsequently disseminated to other parts of the world (Fig. 4*E*).

### Coregulation of Higher Oil Content by *TT8s* with *STKs* in Yellow Seeds of *B. juncea*.

A clear gap in our knowledge is why yellow seeds accumulate more oil. To provide insights into this phenomenon, we first assessed SOC across a panel of 480 *B. juncea* accessions grown under four contrasting environments (20). The SOC was positively correlated between accessions in different environments (*r*, 0.38 to 0.58; *SI Appendix*, Fig. S9), such that the broad-sense heritability of SOC reached 0.59 (Dataset S25). The SCP was measured in 271 *B. juncea* accessions grown in Guiyang in 2018 and ranged from 6 to 27% (Dataset S25). The yellow-seeded accessions had higher SOC, heavier seed weight, and lower SCP than the black-seeded ones in *B. juncea* (*SI Appendix*, Fig. S10 and Dataset S26). SOC was positively correlated with seed color (*r*, 0.34 to 0.44), and negatively correlated with SCP (*r*, −0.36 to −0.48; *SI Appendix*, Fig. S9). The thousand seed weight (TSW) was also positively correlated with seed color (*r*, 0.35 to 0.43), and negatively correlated with SCP (*r*, −0.27 to −0.37; *SI Appendix*, Fig. S9).

**Fig. 5.**
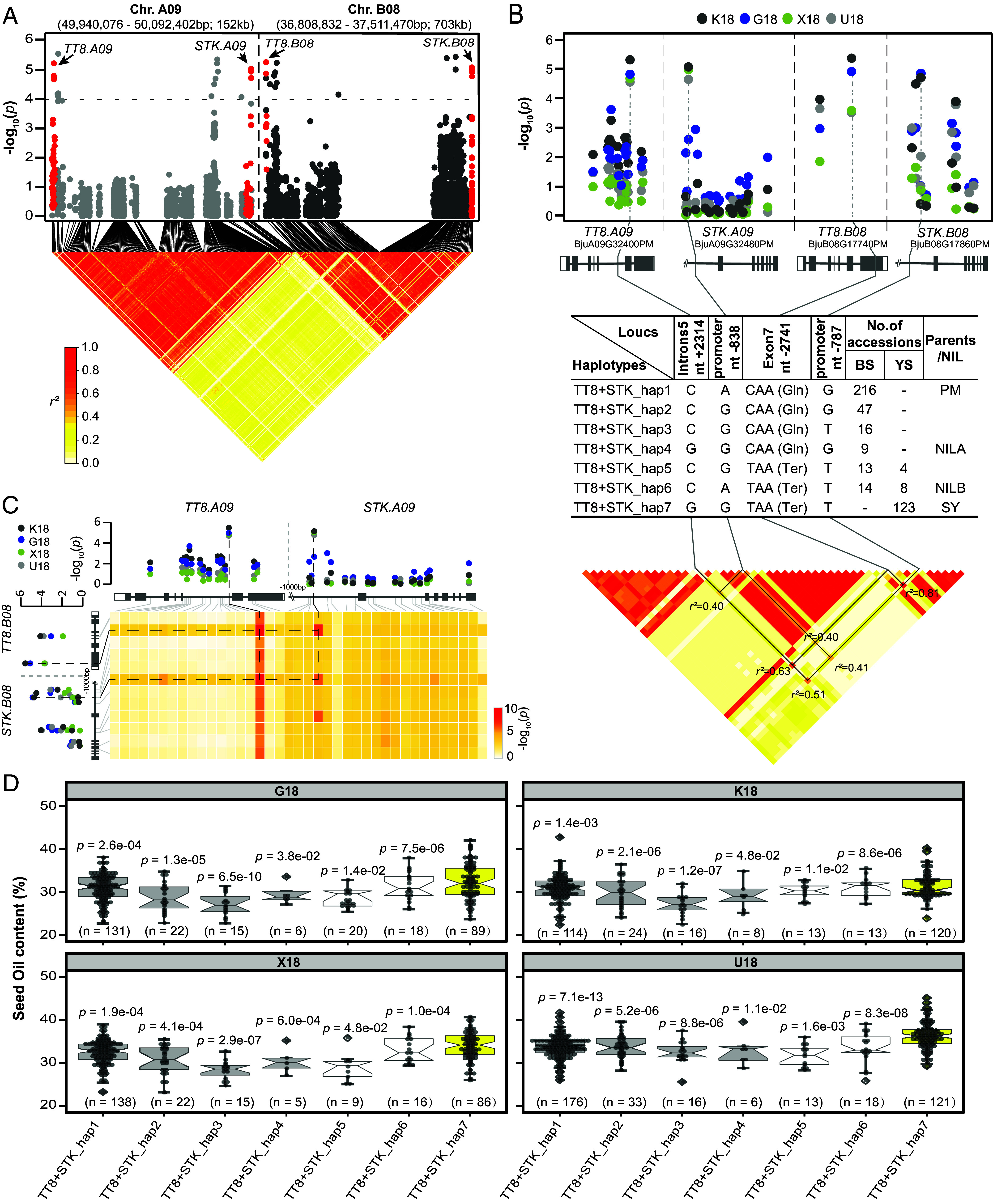
Association analysis of SOC as related to chromosomes A09 and B08 in *B. juncea.* (*A*) Manhattan plot showing two haplotypes (49,940,076 to 50,092,402 bp and 36,808,832 to 37,511,470 bp on chromosomes A09 and B08, respectively) were significantly associated with oil content. The gray dotted line indicates the significance threshold (−log_10_(*P*) = 4.0). The red plots represent the position of these SNPs in two homologous *TRANSPARENT TESTA* (*TT8*) and *SEEDSTICK* (*STK*) genes on chromosomes A09 and B08. Heatmaps showed strong LD between these SNPs, particularly in the *TT8* and *STK* gene regions. (*B*) The SNP each located in intron 5 of *TT8.A09*, the promoter of *STK.A09*, exon 7 of *TT8.B08*, and the promoter of *STK.B08* exhibited significant association with oil content. Seven haplotypes with frequency greater than 0.01 were identified in the *TT8* and *STK* gene regions. (*C*) The four SNP–SNP interaction pairs were detected in *TT8* and *STK* gene regions. (*D*) Boxplots for oil content based on the haplotypes for two homologous *TT8* and *STK* genes under four different environments. Box edges represent the 0.25 quantile and 0.75 quantile with the median values shown by bold lines. Whiskers extend to data no more than 1.5 times the interquartile range, and the remaining data are indicated by dots. G18: Guiyang, China, 2018. X18: Xiangtan, Hunan, China, 2018. K18: Kunming, China, 2018. U18: Urumqi, China, 2018. *P*-values were calculated with a two-tailed Student’s *t* test.

Next, using the GWAS, we associated 55 and 21 candidate genes with SOC (*SI Appendix*, Fig. S11 and Dataset S27) and SCP (*SI Appendix*, Fig. S12 and Dataset S28), respectively. As expected, the SNPs significantly associated with seed color in the *TT8s* were also markedly correlated with SOC, SCP, and TSW (Fig. 5*A* and *SI Appendix*, Figs. S13*A* and S14*A*). Surprisingly, an SNP (A→G, nt -838) located in the promoter of *STK.A09* (BjuA09G32480PM), and an SNP (G→T, nt -787) in the promoter of *STK.B08* (BjuB08G17860PM) were also significantly associated with SOC, SCP, and TSW (Fig. 5*B* and *SI Appendix*, Figs. S13*B* and S14*B*). These four SNPs in *TT8s* and *STKs* appear to have fixed, and SNP–SNP interaction analysis detected their strong interaction in the regulation of SOC, SCP, and TSW (Fig. 5*C* and *SI Appendix*, Figs. S13*C* and S14*C*). *STKs* coevolved with *TT8s* (*SI Appendix*, Fig. S15) and formed seven TT8 + STK haplotypes in *B. juncea* (Fig. 5*B* and *SI Appendix*, Figs. S13*B* and S14*B*). Compared with the other haplotypes, TT8 + STK_Hap 7 accessions produce yellow seeds with the highest SOC, the lowest SCP, and the heaviest TSW (Fig. 5*D* and *SI Appendix*, Figs. S13*D*, S14*D*, and S16, and Dataset S29). Both NILs (TT8 + STK_Haps 5 and 6), because of breakdown of tight linkage of TT8 + STK_Hap 7, exhibited lower oil content, lighter seed weight, and thicker seed coat than their recurrent parent SY although they synchronically flowered (*SI Appendix*, Fig. S17). These results suggest that *TT8s* and *STKs* coregulate these traits.

**Fig. 6.**
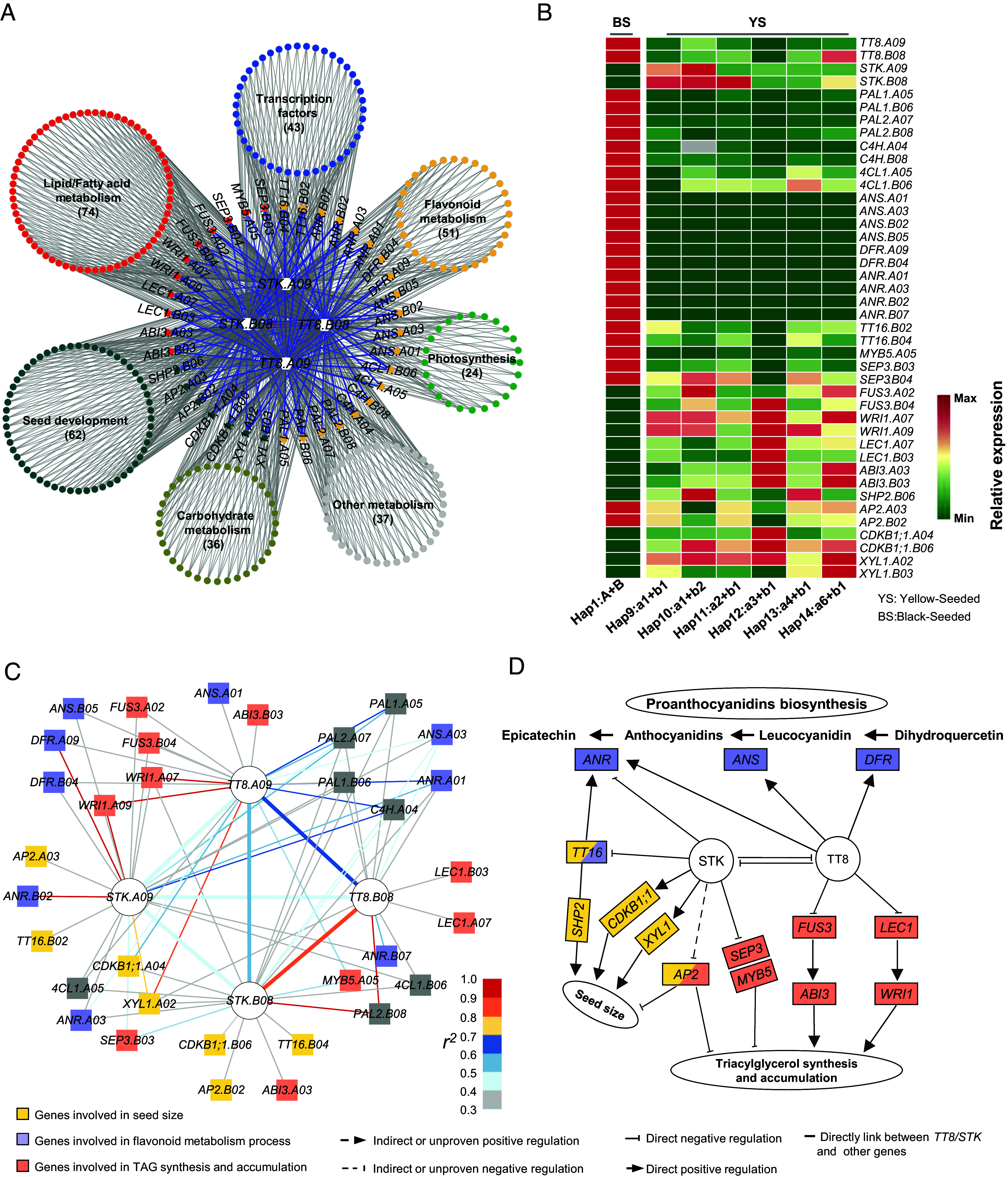
The molecular regulatory network of *TT8* and *STK* genes involved in seed coat and oil content in *B. juncea*. (*A*) Coexpression network of *TRANSPARENT TESTA 8* (*TT8*) and *SEEDSTICK* (*STK*) genes. Based on the functional annotation, coexpression networks were classified into the following groups: lipid/fatty acid metabolism (red nodes), seed development (dark cyan nodes), flavonoid metabolism (gold nodes), transcription factors (blue nodes), carbohydrate metabolism (khaki nodes), and photosynthesis (green nodes). Genes in each category in close linkage with *TT8* and *STK* genes are indicated. (*B*) Expression analysis of the genes for flavonoid biosynthesis, oil content, and seed weight in the seed coat 15 d after pollination in the wild-type and six mutated haplotypes. (*C*) Association networks were constructed between *TT8s*/*STKs* and other genes by LD. (*D*) A proposed model for the function of *TT8* and *STK* genes in the regulation of seed color, seed weight, and oil content.

To further dissect how the genes *TT8s* and *STKs* coordinate to regulate these traits, we used transcriptome data of seeds and their testa from two accessions for each haplotype, at three developmental stages to construct coexpression networks. Twenty-five and twenty-six gene modules were identified for seed and testa, respectively (*SI Appendix*, Fig. S18). The *TT8s* and *STKs* fell in a blue module for seed coats and orange-red module for seeds that showed a significant positive correlation with seed color (*r* = 0.47; *r* = 0.35; *SI Appendix*, Fig. S18). A KEGG analysis showed that the genes from both modules were involved in flavonoid biosynthesis, lipid metabolism, glycosyltransferases, and carbohydrate metabolism, among others (*SI Appendix*, Fig. S19). Deconstruction of the blue and the orange-red module revealed *TT8s* and *STKs* directly linked to 332 and 345 additional genes, including 55 and 52 genes associated with flavonoid metabolism, and 74 and 85 genes involved in lipid/fatty acid metabolism, respectively (Fig. 6*A*, *SI Appendix*, Fig. S20*A*, and Dataset S30).

Mechanistically, upregulation of *STK*s in the yellow-seeded mutants compared to the black-seeded *B. juncea* accessions suggests *TT8s* and *STKs* inhibit each other during seed development (Fig. 6*B* and *SI Appendix*, Figs. S20*B*, S21, and S22). In seeds and their testa of yellow-seeded mutants the up-regulated *STKs*, directly and indirectly, inhibited the expression of negative transcriptional regulators *SEPALLATA3*(*SEP3*), *MYB DOMAIN PROTEIN 5* (*MYB5*) (49), and *APETALA 2* (*AP2*) (50) to promote the oil accumulation, respectively (Fig. 6*D* and *SI Appendix*, Figs. S20*C*, S23, and S24). Additionally, the *tt8* mutations enhanced expression of the positive master regulators *LEAFY COTYLEDON 1* (*LEC1*) and (*FUS3*) (51–53) for oil biosynthesis (Fig. 6*D* and *SI Appendix*, Figs. S20*C*, S23, and S24). These transcriptional regulators eventually caused upregulation of 66 genes for fatty acid/lipid synthesis including *DIACYLGLYCEROL ACYLTRANSFERASE 1* (*DGAT1*), *3-KETOACYL-ACYL CARRIER PROTEIN SYNTHASE III* (*KASIII*), *3-KETOACYL-COA SYNTHASE 4* (*KCS4*), and *BIOTIN CARBOXYL CARRIER PROTEIN 1* (*BCCP1*) and therefore promoted seed lipid synthesis (*SI Appendix*, Fig. S25). Meanwhile, in the yellow-seeded mutants, the up-regulated expression of *STK* promoted the expression of *CYCLIN-DEPENDENT KINASE B1;1* (*CDKB1;1*) (54), *SHATTERPROOF 2* (*SHP2*) (55), and *ALPHA-XYLOSIDASE 1* (*XYL1*) (56), and inhibited the expression of *TRANSPARENT TESTA16* (*TT16*) (57) and *AP2* (50) to promote seed development (Fig. 6*D* and *SI Appendix*, Figs. S20*C*, S23, and S24). In addition, artificial and natural selection has resulted in the core genes *TT8s* and *STKs* closely linked to other genes in the network (Fig. 6*C*). In summary, antagonistic *TT8* and *STK* form a transcriptional regulatory network that regulates higher seed oil accumulation and bigger seed size in yellow-seeded *B. juncea* (Fig. 6*D* and *SI Appendix*, Fig. S20*C*).

## Discussion

The seed color trait is regulated by the gene *TT8* not only in *Brassicaceae* species including *Arabidopsis thaliana* (10), *B. rapa* (58), and *B. juncea* (9, this study) but also in other plant species such as *Oryza sativa* (59), *Ipomoea purpurea* (60), *Pisum sativum* (61), and *Phaseolus vulgaris* (62). The *tt8* mutants produce bright yellow seed and are hence the best target genes for breeding yellow-seeded varieties. For the species *B. juncea* and *B. rapa* with natural yellow-seeded mutants, many yellow-seeded varieties were developed by conventional breeding and commercially released. However, in oilseed crops *B. napus* (53), *Thlaspi arvense* (63), and *Camelina sativa* (64), no spontaneous yellow-seeded mutants have been found up to date and therefore induced mutation or knockouts of *TT8* were used to create yellow-seeded mutants with enhanced oil accumulation. In addition, interspecific crosses were also used to develop yellow-seeded oilseed *B. napus* cultivars (3, 65). We used yellow-seeded *B. juncea* accessions as gene donors to cross with *B. napus*. As a result, pure yellow-seeded *B. napus* cultivars with SOC of over 50% have been developed (66, 67). Besides increased SOC, yellow-colored *Brassica* seeds have significantly lower fiber and higher protein contents (3, 68), which improve nutritional value for humans and livestock (69, 70). The varieties with different classes of seed or grain color have also been selected for intended uses and/or market demands in cereals and legumes such as *O. sativa* (71, 72), *Sorghum bicolor* (73), *P. sativum* (74).

In most studies, the spontaneous yellow seed trait has been shown to be controlled by duplicated recessive loci in *B. juncea*. However, the trait is also reported to be controlled by a single locus in the few studies (75, 76). As shown here, the brown-seeded accessions used in these studies, e.g., Blaze 25-11 (4), carry a mutated *tt8* allele at either locus. In fact, Indian mustard is called brown mustard because of less pigmentation in accessions carrying the *tt8.a1* allele.

*B. juncea* is an allotetraploid from progenitor species *B. rapa* and *B. nigra*. *B. nigra* was primarily grown for condiment and has low genetic divergence. No spontaneous yellow-seeded variants have been discovered in *B. nigra* (2). Wild *B. rapa* was initially domesticated into turnip and/or oilseeds 3,430 to 5,390 y ago (77). Oilseed yellow sarson (*B. rapa ssp. trilocularis*) is characterized by yellow-colored seeds. It may originate in north west of India *c.*1,200 BC (78), which occurred earlier than the yellow-seeded *B. juncea* (2,300 y ago). The yellow seed trait is reported to be controlled by *TT8* (58), *Transparent Testa 1* (*TT1*) (79) or *TRANSPARENT TESTA GLABRA 1* (*TTG1*) (80) in *B. rapa*. However, the *TT8s* are the sole genes for the yellow seed trait in *B. juncea*. All six *tt8.a* mutations are distinguished in *B. juncea* from that in *B. rapa* (9, 58, this study), supporting yellow seed is a domestication trait after speciation of *B. juncea*.

We discovered that *TT8*s comprise with *STKs* haplotype block in *B. juncea*. They inhibited each other in gene expression and coregulate a couple of seed traits including seed color and oil accumulation in *B. juncea* (Fig. 6 and Dataset S30). The identified *STKs* provide new gene resources for genetic improvement of seed traits. Targeted knockout of the *STKs* genes may further decipher their mechanism of interaction with *TT8s* for regulation of seed traits in *B. juncea* mutants of various *tt8* haplotypes.

In conclusion, these findings open broad avenues for targeted breeding of yellow-seeded oilseed crops with elevated oil content.

## Materials and Methods

### Genome Survey, Assembly, and Gap Filling.

The 17-kmers with Jellyfish (v2.3.1) (81) software using the Illumina reads were used to estimate the genome size. Backbone contigs from HiFi reads and ONT reads were assembled using HiFiasm (v0.19.3) (21) with default parameters. HiCanu (v2.2) (23) and Verkko(v1.4) (24)were also used to obtain genome assemblies based on HiFi reads. For ONT assemblies, the NextDenovo software (v2.5.0) (22) with default parameters was used to assemble the long reads into contigs. The contigs were polished to improve the single-base accuracy using NextPolish software (v1.4.1) (25) with three rounds of iteration. The interaction map for performing scaffolding was generated using Juicer (82) software and 3D-DNA pipeline (v180922) (83). RagTag (26) software was used for gap closing. Minimap2 (v2.17) (27) software was used to extend telomeres.

Detailed procedures for the PM genome assessment and genome annotation are provided in *SI Appendix*.

### Structural Variation Analysis.

The two genomes were aligned using Mummer4 (v.4.0.0) (84) with parameters settings “−l 50 −c 100”. The alignment block was then filtered out of the mapping noise and the one-to-one alignment was identified by delta-filter with parameters settings “−r -q i 90 −l 100”. SyRI (85) was used to identify SNPs and SVs with the default parameters.

### Fine Mapping and Positional Cloning.

QTL detection was performed using the composite interval mapping procedure of the software WinQTL Cartographer 2.5 (86). based on the high-density genetic map described in ref. 20. The physical maps of the regions of the target genes were constructed by BAC-by-BAC methods using tightly linked and cosegregating markers. The primers used for *TT8* gene amplification are listed in Dataset S31.

### Plasmid Reconstruction and Plant Hypocotyl Transformation.

The full-length genomic DNA fragments (*TT8.A09*, ~5.4 kb, from −1,931 to + 3,458 bp; *TT8.B08*, ~4.6 kb, from −1,842 to + 2,765 bp) from PM were amplified and inserted into a pCambia1305 binary vector using restriction enzyme sites *Eco*RI and *Sac*I. Then the reconstructed plasmid was separately transformed into hypocotyls of SY seedlings following an *Agrobacterium*-mediated transformation protocol (87). Positive T_0_ transgenic lines were obtained by antibiotic screening and PCR amplification, and then transplanted into pots. The primer sequences used for vector construction and PCR amplification are given in Dataset S31.

### Yeast One Hybrid (Y1H) Assay.

For the Y1H assay, the promoter sequences of *DRFs*, *ANSs*, and *ANRs* were amplified from PM and the fragments were digested with *Sma*I and *Eco*RI, then ligated into pHIS2.1 digested with the same enzymes to construct bait plasmids. The full-length cDNA sequences of *TT8s* were cloned from SY and PM separately and digested with restriction enzymes *Xma*I and *EcoR*I, then ligated into pGADT7 digested with the same enzymes to construct prey plasmids.

### GWAS Analysis.

A total of 4,529,618 SNPs with MAF ≥ 0.05 and missing rate ≤ 0.1 in the population were used for the GWAS using the genome-wide efficient mixed model association program (GEMMA) (88) under a mixed-linear model. Significant *P*-value thresholds [*P* < 10^−6^ for seed color, and *P* < 10^−4^ for SOC, SCP, and TSW, respectively] were set to control the genome-wide type I error rate. SNP–SNP interactions between *TT8* and *STK* genes were examined by the R package “SIPI” (89).

### Selective Sweep Analysis.

Fixation indices (*F_st_*values) were calculated by VCFtools with parameters and settings “--fst-window-size 10,000 --fst-window-step 5,000” (90). The top 5% of regions were assigned as candidate selective regions, and genes in these regions were considered as candidate genes.

### Haplotype Network Analysis.

Haplotype analysis for *TT8* genes has been carried out considering SNPs and InDel marker in 1,002 *B*. *juncea* accessions. Haplotype networks were constructed and visualized using the PopART software (91).

### Phylogenetic Analysis and Divergence Time Estimation.

A maximum likelihood phylogeny was inferred by IQ-TREE (v 1.6.12) (92) with concatenated alignments and the best-fitting model, and with 1,000 bootstrap replicates. To infer divergence time, we used MCMCTree in PAML 4 (93) under a relaxed-clock model (correlated molecular clock) with approximate likelihood calculation and maximum likelihood estimation of branch lengths. We constrained the root age to 8,000 to 14,000 y (20) and performed 10,000 samplings with “burnin=50,000” and “sampfreq=50”.

### Metabolite Analysis.

Metabolites were extracted from the seed coat after separation of the seed coat from the rest of the seed. Detailed descriptions of metabolites analysis are shown in *SI Appendix*.

### Weighted Gene Coexpression Network Analysis (WGCNA).

Gene expression data were obtained from the seed coat transcriptomes of the 14 *B. juncea* accessions. We used the R package “WGCNA” (94) to construct coexpression networks with a cutoff of the weight parameter set at 0.2. Network visualization for each module was carried out using the Cytoscape software version 3.6 (95).

## Supplementary Material

Appendix 01 (PDF)

Dataset S01 (XLSX)

Dataset S02 (XLSX)

Dataset S03 (XLSX)

Dataset S04 (XLSX)

Dataset S05 (XLSX)

Dataset S06 (XLSX)

Dataset S07 (XLSX)

Dataset S08 (XLSX)

Dataset S09 (XLSX)

Dataset S10 (XLSX)

Dataset S11 (XLSX)

Dataset S12 (XLSX)

Dataset S13 (XLSX)

Dataset S14 (XLSX)

Dataset S15 (XLSX)

Dataset S16 (XLSX)

Dataset S17 (XLSX)

Dataset S18 (XLSX)

Dataset S19 (XLSX)

Dataset S20 (XLSX)

Dataset S21 (XLSX)

Dataset S22 (XLSX)

Dataset S23 (XLSX)

Dataset S24 (XLSX)

Dataset S25 (XLSX)

Dataset S26 (XLSX)

Dataset S27 (XLSX)

Dataset S28 (XLSX)

Dataset S29 (XLSX)

Dataset S30 (XLSX)

Dataset S31 (XLSX)

## Data Availability

The genome assembly and gene annotation for *B. juncea* var. PM were deposited at the China National Genomics Data Center with BioProject ID PRJCA030880 (96). The genome sequence data for *B. juncea* var. SY and the resequencing data for 480 *B. juncea* accessions are accessible under NCBI BioProject No. PRJNA615316 (97). The RNA-seq data of seed and seed coat of 63 and 45. *B*. *juncea* inbred lines used in this study have been deposited in the NCBI BioProject Nos. PRJNA1185494 (98) and PRJNA1173242 (99), respectively. All other data are included in the manuscript and/or supporting information.
