# Supplementary material for: Natural variations in TT8 and its neighboring STK confer yellow seed with elevated oil content in Brassica juncea
Source: Proc Natl Acad Sci U S A. 2025 Jan 30;122(5):e2417264122. doi: 10.1073/pnas.2417264122 (PMC11804580; doi:10.1073/pnas.2417264122)
Supplement: Supplementary file 1 — Appendix 01 (PDF) [file pnas.2417264122.sapp.pdf]

Supporting Information for

Natural variations in *TT8* and its neigboring *STK* confer yellow seed with elevated oil content in *Brassica juncea*

Lunwen Qian[1,2#], Liu Yang[1,2#], Xianjun Liu[1,8#], Tianyi Wang[9,#], Lei Kang[1, 2], Hao Chen[1, 2], Yin Lu[1, 2], Yukun Zhang[1,2], Shujie Yang[1,2], Liang You[1,2], Min Yao[1,2], Xingru Xiang[1,2], Kan Cui[3], Ying Guo[3], Bin Yang[1,11], Mingli Yan[1,10], Shitou Xia[3], Jinling Meng[5], Tao Lin[4], Annaliese S. Mason[6], Rod J. Snowdon[7], and Zhongsong Liu[1,2]*.

[1]College of Agronomy, Hunan Agricultural University, Changsha 410128, China

[2]Yuelushan Laboratory, Changsha 410128, China

[3]Hunan Provincial Key Laboratory of Phytohormones and Growth Development, College of Bioscience and Biotechnology, Hunan Agricultural University, Changsha 410128, China

[4] College of Horticulture, China Agricultural University, Beijing 100193, China

[5]National Key Laboratory of Crop Genetic Improvement, Key Laboratory of Rapeseed Genetic Improvement, Ministry of Agriculture China, Huazhong Agricultural University, Wuhan, China

[6]Plant Breeding Department, The University of Bonn, Katzenburgweg 5, 53115, Bonn, Germany

[7]Department of Plant Breeding, Justus Liebig University Giessen, Heinrich-Buff-Ring 26-32, Giessen 35392, Germany

[8]Present address: College of Agriculture and Biology, Hunan University of Humanities, Science and Technology, Loudi, Hunan 417000, China

[9]Smartgenomics Technology Institute, Tianjin 301700, China

[10]Present address: Crop Research Institute, Hunan Academy of Agricultural Sciences, Changsha, Hunan 410125, China

[11]Present address: Guizhou Institute of Oil Crops, Guizhou Academy of Agricultural Sciences, Guiyang 550000, China

Materials and Methods

Genome assessment. We aligned the A (CentBrs, CRB and TR805) (1, 2) and the B (CRB, pBNBH35 and CLs) (3-5) subgenome centromeric repeat sequences to the PM genome assembly using BLAST (6) (*E*-value 1e-5) and the densities of centromeric repeat sequences were calculated to indentify the centromere regions. Tidk (https://github.com/tolkit/telomeric identifier) was used to identify the telomeres with the parameters "--minimum 5 --maximum 12". HiFi reads were mapped using the Minimap2 (v2.17) (7). SAMtools (v1.14) (8) was used to calculated genome coverage and mapping rate. BUSCO (v5.1.2) (9) was used to evaluate the integrity of the genetic region with 1,614 core plant conserved genes. The repeat sequences of the genome were assessed using the LTR Assembly Index (LAI) (10).

Genome annotation. LTR_FINDER (11), RepeatScout (http://www.repeatmasker.org/), and RepeatModeler (http://www.repeatmasker.org/RepeatModeler.html) were used to construct *de novo* repeat sequence library. The *de novo* and RepBase database (http://www.girinst.org/repbase) libraries were merged, and RepeatMasker (http://repeatmasker.org/) was used to annotate the repeat sequences based on this merged library.

A comprehensive strategy combing *ab initio* prediction, protein-based homology searches, and RNA sequencing was used to annotate the gene structure. Protein sequences from ten genomes were used for homology-based gene annotation. TBLASTn (v2.2.26) (6) and GeneWise (v2.4.1) (12) were used to predict the gene structure of the BLAST hits. Augustus (v3.4.0) (13), SNAP (14), GlimmerHMM (v3.0.4) (15), GENEID (16) and GENSCAN (17) were used for *de novo* gene prediction. Trinity (18) was used for *de novo* transcript assembly, and the result was used for transcript annotation using PASA (19). EVidenceModeler (EVM) (v2.0.0) (20) was used to integrate these prediction results into weighted consensus gene structures.

All protein-coding genes were aligned to two integrated protein sequence databases: SwissProt (https://ftp.uniprot.org/pub/databases/uniprot/current_release/knowledgebase/complete/uniprot_sprot.fasta.gz) and NR (ftp://ftp.ncbi.nlm.nih.gov/blast/db/FASTA/nr.gz). Protein domains were annotated by InterProScan (21). The Gene Ontology (GO) terms for each gene were obtained from the corresponding InterProScan entry. The pathways in which the genes might be involved were assigned by BLAST (6) against the KEGG databases (https://www.genome.jp/kegg/brite.html), with an E-value cutoff of 1e-5. Functional annotation results were merged from above two strategies.

The softwares tRNAscan-SE (22) and INFERNAL (23) were used to annotate genes for tRNA and for miRNA and snRNA, respectively. rRNA sequences were predicted by aligning to *Arabidopsis* template rRNA sequences.

Plant materials and phenotyping. The geographic origin, subspecies classification and seed color of the 480 re-sequenced *Brassica juncea* accessions are shown in (Dataset S12) (24). These accessions were planted in the field in Guiyang (G18; E106.72/N026.58), in Xiangtan (X18; E112.90/N027.86), in Kunming (K18; E102.72/N025.04) and in Urumqi (U18; E087.60/N043.80). Each accession was grown in a three-row plot, with 15 plants per row, 5 cm between plants within each row and 15 cm between the rows. A set of 172 recombinant inbred lines (RILs) were derived from the cross Sichuan Yellow (SY) × Purple Leaf Mustard (PM), and were planted in the field in Xiangtan (X18; E112.90/N027.86), and grown in 2.5 m rows, spaced 0.5 m apart, with 12 individuals in each row. The transgenic lines were planted in a greenhouse in Changsha, China. To avoid the effect of environment, we planted these transgenic materials and controls in the same greenhouse. The seed color was scored visually and by vanillin staining described by Lu et al. (25).

Fine mapping and positional cloning. A 1,000-permutation test was performed to estimate a significance threshold of the test statistic for a QTL based upon a 5 % experiment-wise error rate (26). The BC_8F_2 mapping populations were constructed using SY as a recurrent parent (*SI Appendix*, Fig. S4), and consisted of 5,434 and 6,537 individual plants segregating only at a single seed color locus. DNA markers were used for screening recessive recombinants (Dataset S15). The primers used are listed in Dataset S16. At the same time, the BAC library was constructed from PM as described by Liu et al. (27). The physical maps of the regions of the target genes were constructed by BAC-by-BAC methods using tightly-linked and co-segregating markers. The primers used for *TT8* gene amplification are listed in Dataset S31.

Plasmid reconstruction and plant hypocotyl transformation. Positive T_0 transgenic lines were obtained by antibiotic screening and then transplanted into pots. When these positive T_0 transgenic lines grew up to the 6-8th true leaf stage in the growth room under the conditions of 16 h day (23°C) / 8 h night (21°C), leaves from the individuals were collected and genomic DNA was extracted by the CTAB method. Presence of the transgene in these lines was further verified by

specific PCR primers.

Yeast one hybrid (Y1H) assay. The full-length cDNA sequences of *TT8s* were cloned from SY and PM separately and digested with restriction enzymes *Xma*I and *Eco*RI, then ligated into pGADT7 digested with the same enzymes to construct prey plasmids. The reconstructed vectors were co-transformed into yeast Y187 (MATa; ura3–52; his3–200; ade2–101; trp1–901; leu2–3, 112; gal4D; gal80D; met–; URA3::GAL1 UAS-GAL1 TATA-LacZ; MEL1; Clontech). The DNA-protein interactions were determined by the growth of the transformants on SD/-Leu/-Trp/-His plates supplied with different concentrations of 3-AT.

Phylogenetic analysis and divergence time estimation. A maximum likelihood phylogeny was inferred by IQ-TREE (v 1.6.12) (28) with concatenated alignments and the best-fitting model, and with 1,000 bootstrap replicates. To infer divergence time, we used MCMCTree in PAML 4 (29) under a relaxed-clock model (correlated molecular clock) with approximate likelihood calculation and maximum likelihood estimation of branch lengths. First, the phylogenetic tree and the coding sequence alignments were imported into baseml to roughly estimate the substitution rate using the GTR + G model. Then MCMCtree was run for the first time to estimate the gradient and Hessian matrix. The resulting file, out.BV, was then used for the final run of MCMCTree to perform approximate likelihood calculations.

Transcriptome analysis. Total RNA was isolated from the seed coat with three biological replicates at 15, 25 and 35 DAP to profile expressed genes.Total RNA was isolated from the seed coat with three biological replicates at 15, 25 and 35 days after pollination (DAP) to profile expressed genes. As above, RNA-seq libraries were constructed and sequenced on an Illumina X Ten platform. The clean reads were mapped against the SY genome using TopHat software (v. 2.0.12) (30). The number of reads mapped was counted using HTSeq software (v.0.6.1) (31) and then FPKM was calculated for each gene. Transcripts less than one per million mapped reads were ignored. Analysis of differential gene expression between two samples was performed using the DESeq R package (v. 1.18.0) (32). Genes with an adjusted p-value < 0.05 found by DESeq were assigned as differentially expressed.

Gene ontology analysis. Gene expression data were obtained from the seed coat transcriptomes of the fourteen *B. juncea* accessions. Gene Ontology (GO) enrichment analysis was completed by OmicShare tools (http://www.omicshare.com/tools) and visualized using the R package "ggplot2" (33). The threshold of significantly enriched GO terms was set to false discovery rate (FDR) < 0.05.

Quantitative RT-PCR analyses. Expression levels of genes involved in the phenylpropanoid metabolism, lignin biosynthesis pathways, seed development and lipid metabolism were quantified by real-time quantitative RT-PCR (qPCR). Total RNAs isolated from the seed coat with three biological replicates at 15, 25 and 35 DAP were reverse-transcribed using M-MLV reverse transcriptase (Promega, USA) and oligo (dT) 15 primer, according to the manufacturer's instructions. The expression level of the *B. juncea UBQ9* gene was used as a loading control (34). Each set of experiments was repeated three times, and the relative quantification method ($2^{-\Delta\Delta CT}$) used to evaluate quantitative variation. The primer sequences for qPCR are listed in Dataset S31.

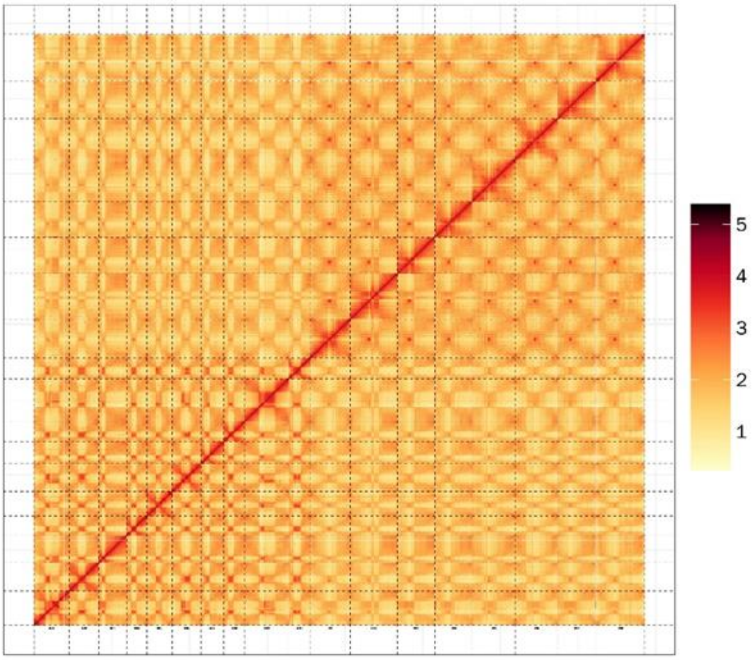

Fig. S1. Hi-C interaction.

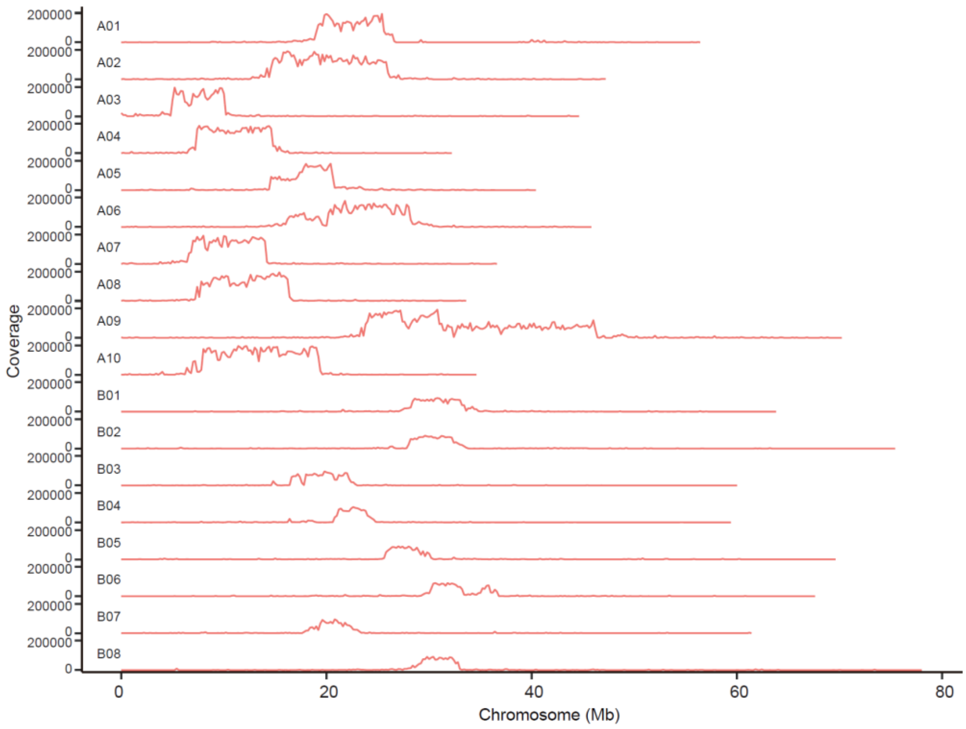

Fig.S2. Identification of the centromere regions based on the alignment of centromeric repeat sequences.

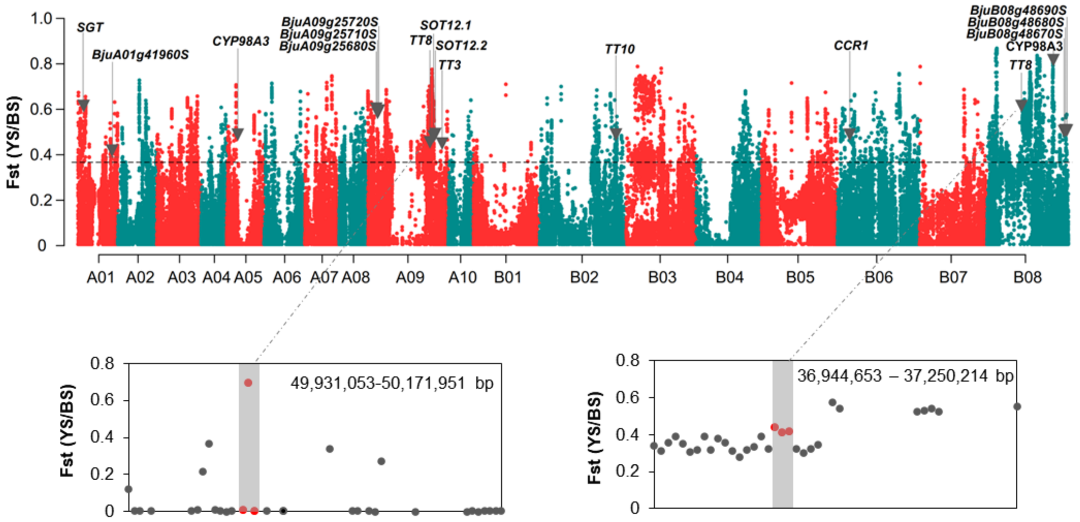

Fig. S3. Genome-wide distribution of selective sweeps identified through comparisons between yellow-seeded (YS) or black-seeded (BS) *Brassica juncea* accessions using F_{st} (sliding window = 10 kb, step = 1kb).

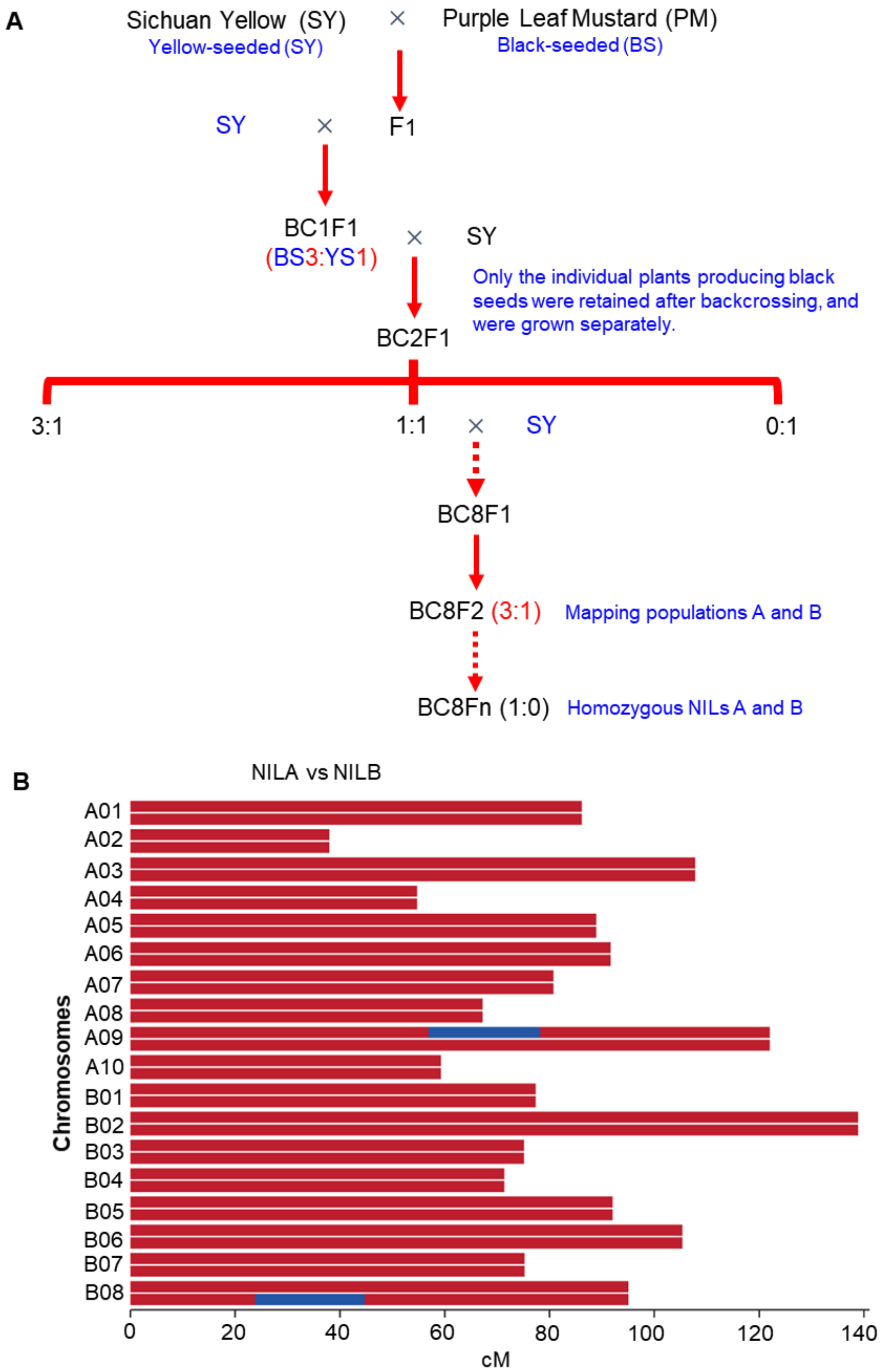

Fig. S4. Development of recombinant inbred lines (RILs) and mapping population through crossing a yellow-seeded parent Sichuan Yellow (SY) with a black-seeded Purple Leaf Mustard (PM). (*A*) Construction of near isogenic lines (NILs) and mapping population. (*B*) Graphical genotypes of near isogenic line A and B (NILA and NILB) on chromosome A09 and B08 show each line carrying a defined introgression chromosomal segment. The blue bars represent the genomic regions of A09 and B08 introgression segments from SY.

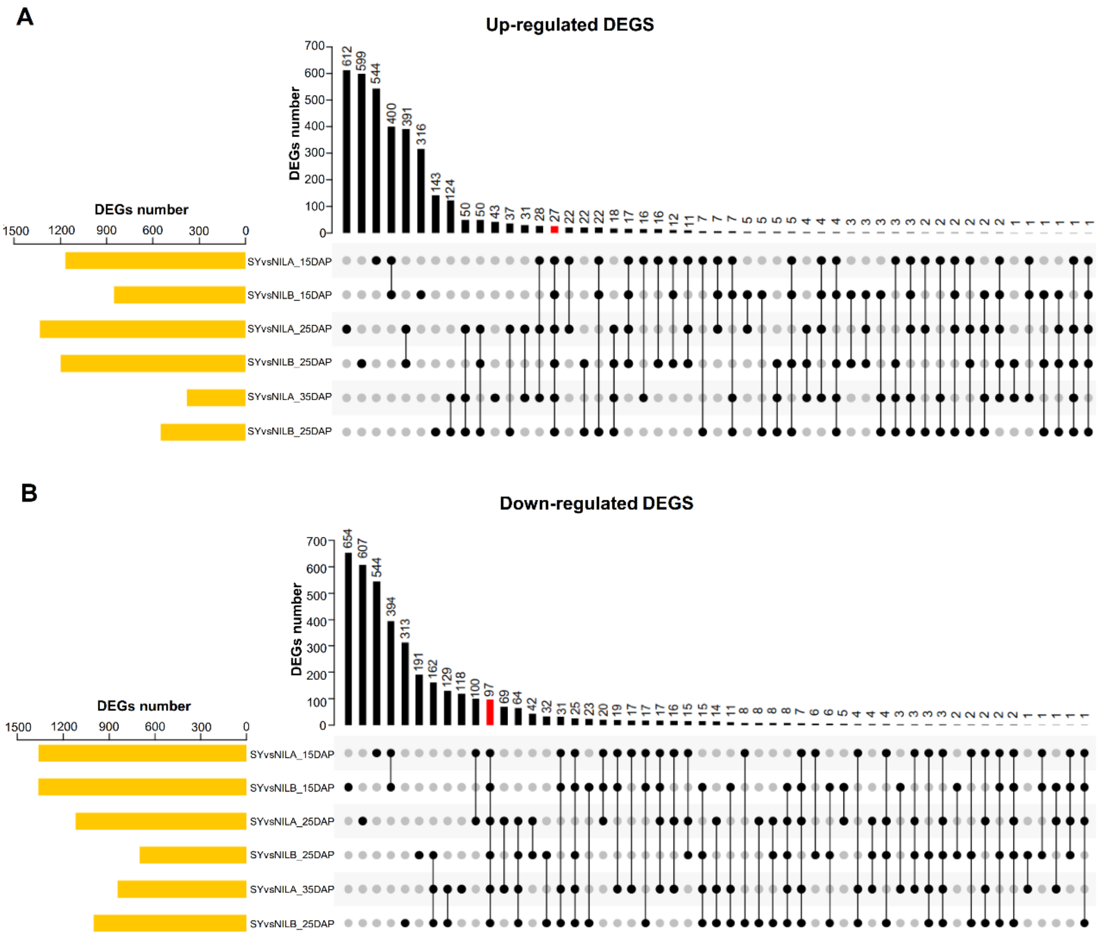

Fig. S5. Expression analysis of differentially expressed genes (DEGs) between Sichuan Yellow (SY) and near isogenic line A and B (NILA and NILB) at 15, 25, and 35 days after pollination (DAPs) in the seed coat of *Brassica juncea*. (*A*) 27 up-regulated DEGs in SY. (*B*) 97 down-regulated DEGs in SY.

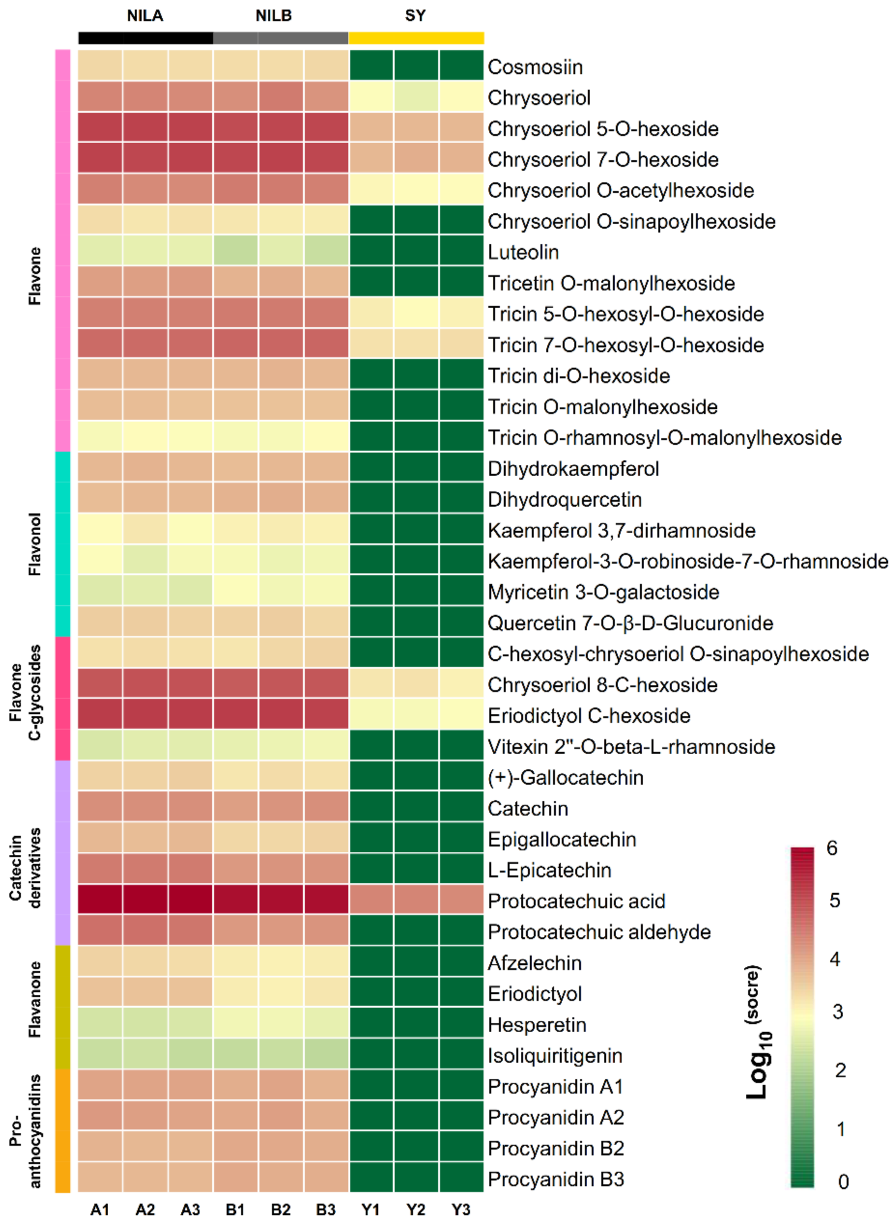

Fig. S6. Heatmap showing 37 different flavonoids in yellow-seeded *Brassica juncea* parent Sichuan Yellow (SY) and in its near isogenic lines A and B (NILA and NILB) derived from crossing with black-seeded Purple Leaf Mustard (PM).

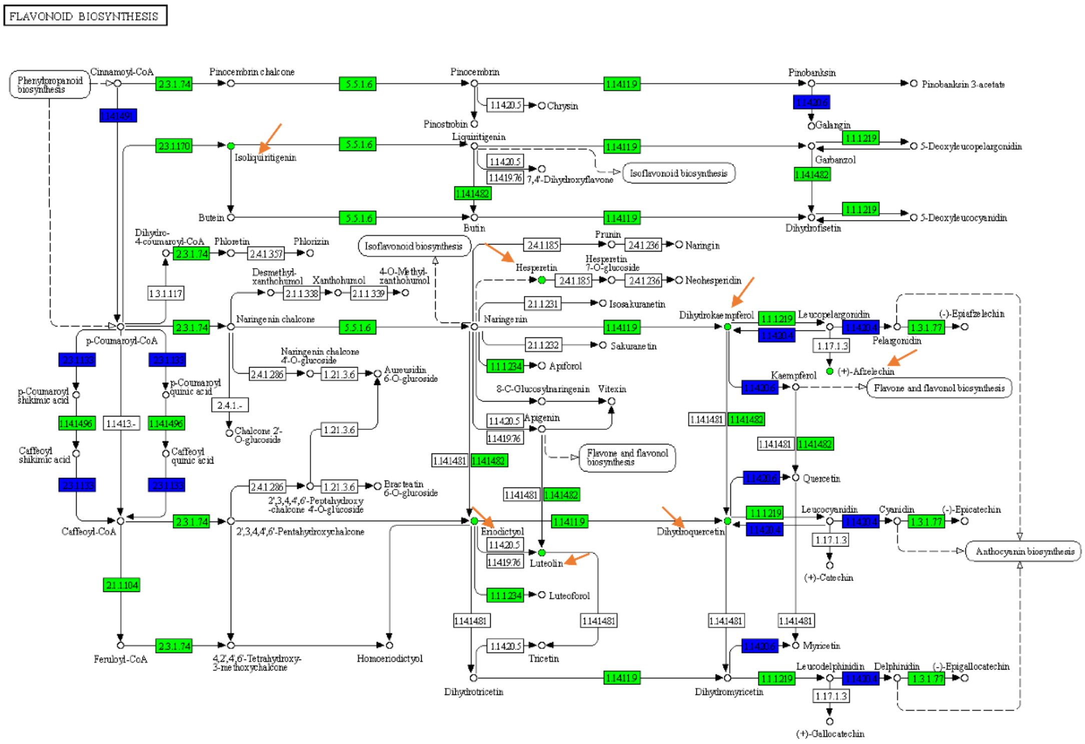

Fig. S7. Flavonoid biosynthesis pathway. The yellow arrows indicate the components involved in seed coat pigmentation of *Brassica juncea.*

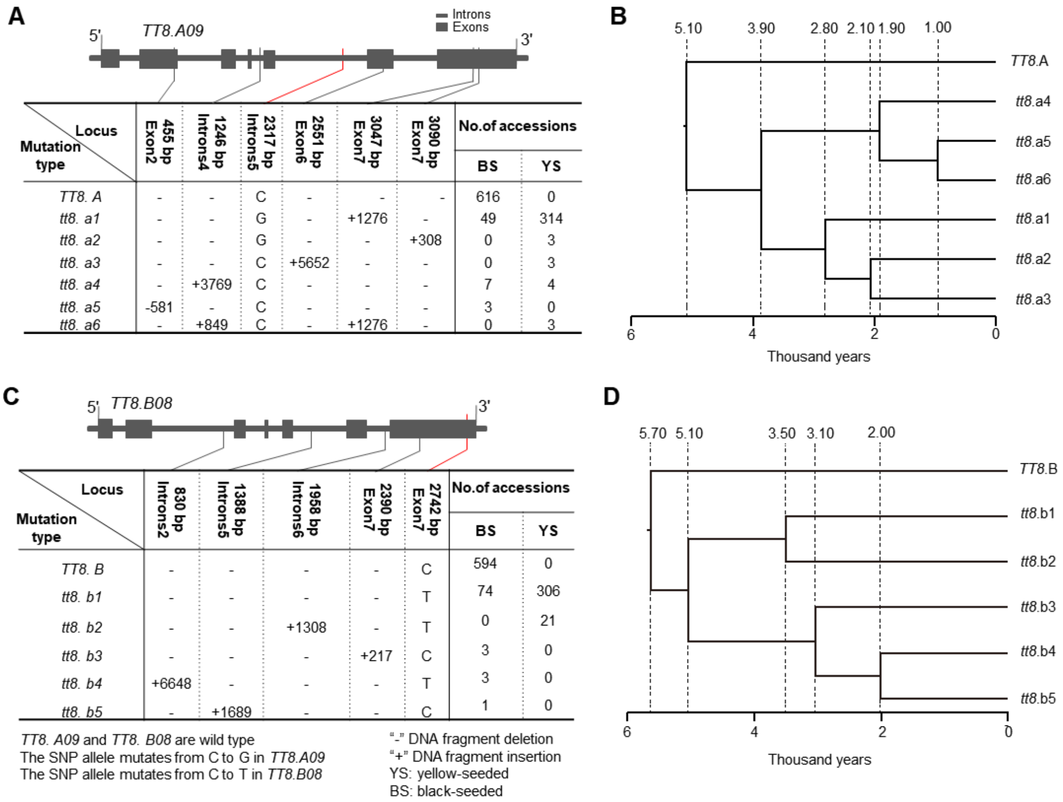

Fig. S8. Allelic variation of *TRANSPARENT TESTA 8* (*TT8*) genes and their evolutionary relationship in 1,002 *Brassica juncea* accessions. Analysis of allelic variation and corresponding phenotypic analysis were performed for *TT8.A09* (*A*) and *TT8.B08* (*C*), respectively. (*B* and *D*) the evolution of these allelic variants.

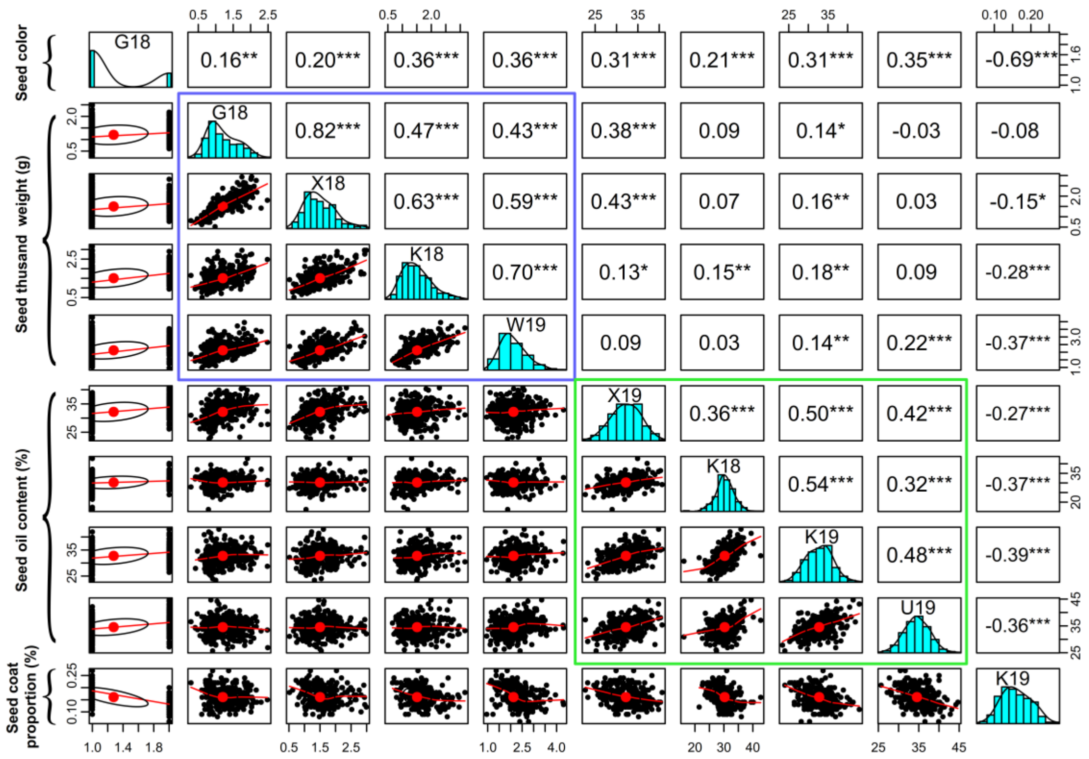

Fig. S9. Correlation coefficients and frequency distributions for seed color, seed oil content, thousand seed weight, and seed coat proportion in 480 *Brassica juncea* accessions. G18: Guiyang, China, 2018. X18: Xiangtan, Hunan, China, 2018. K18: Kunming, China, 2018. U18: Urumqi, China, 2018. (*$P \leq 0.05$, **$P \leq 0.01$, ***$P \leq 0.001$)

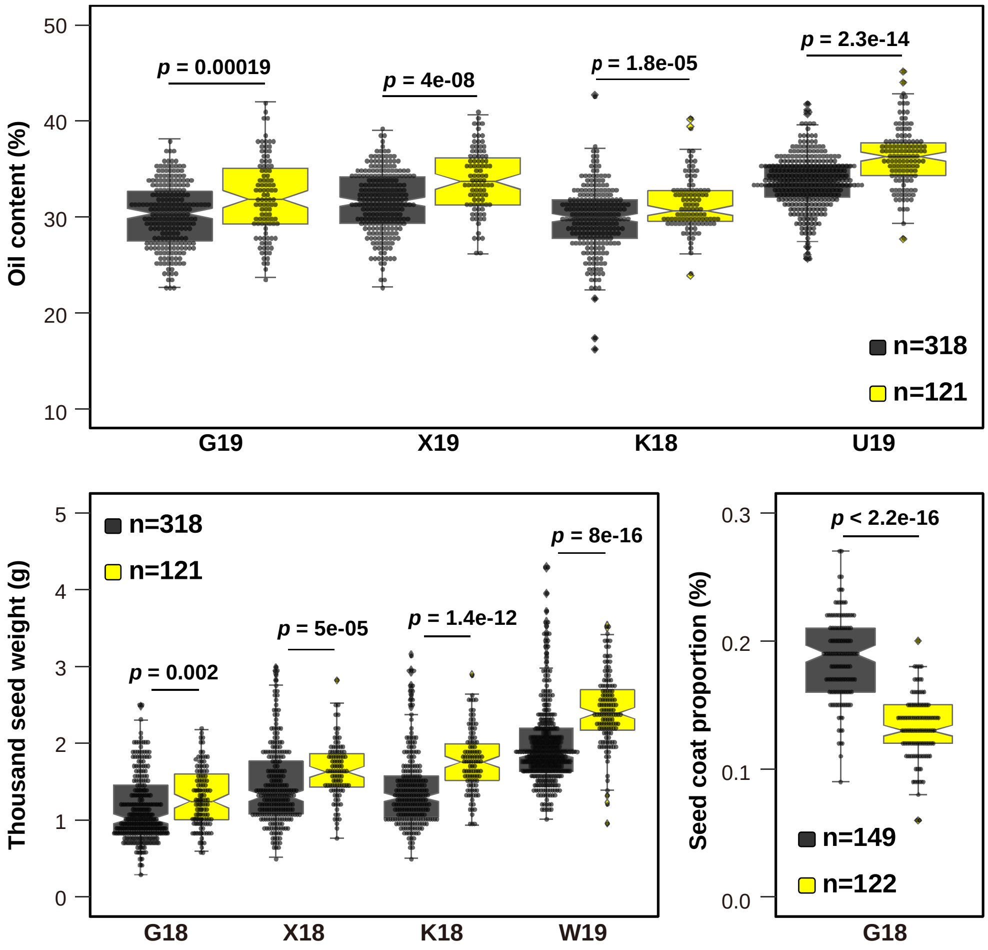

Fig. S10. Comparative analysis of seed oil content, thousand seed weight and seed coat proportion in yellow- and black- seeded accessions of *Brassica juncea*. G18: Guiyang, China, 2018. X18: Xiangtan, Hunan, China, 2018. K18: Kunming, China, 2018. U18: Urumqi, China, 2018. *p*-value was calculated using two-sided *t*-test.

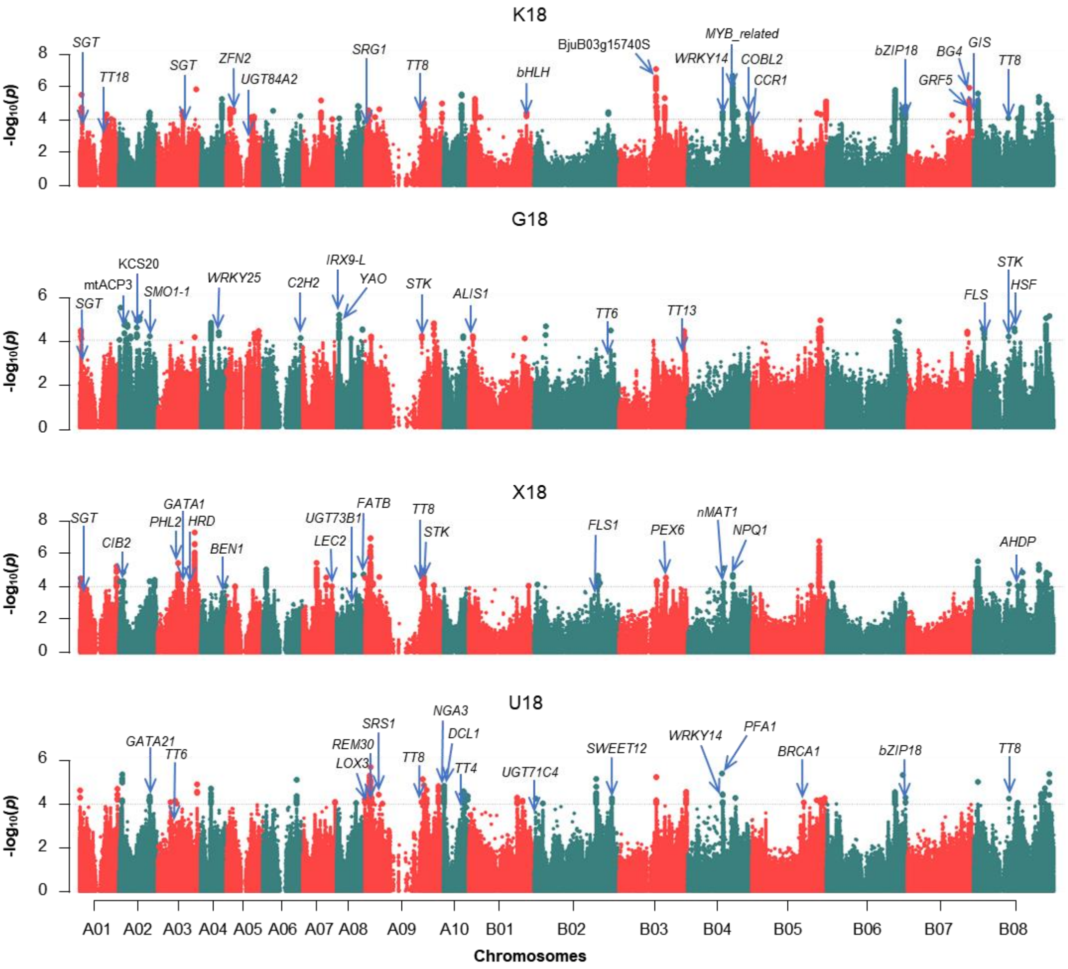

Fig. S11. GWAS analysis of oil content in a panel of *Brassica juncea* accessions in four different environments. Significantly associated seed oil content candidate genes are labeled in each environment. The significance threshold of -log$_{10}$(p) value was set at 4. G18: Guiyang, China, 2018. X18: Xiangtan, Hunan, China, 2018. K18: Kunming, China, 2018. U18: Urumqi, China, 2018.

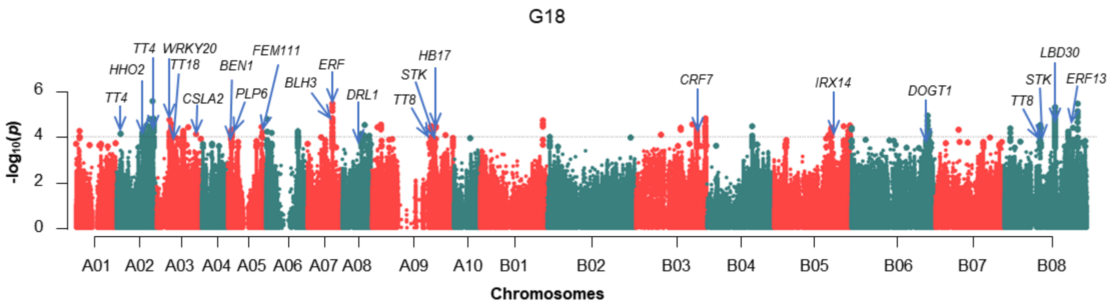

Fig. S12. GWAS analysis of seed coat proportion in a set of *Brassica juncea* in Guiyang in 2018 (G18). Candidate genes significantly associated with seed coat proportion are labeled. The significance threshold of -$\log_{10}(p)$ value was set at 4.

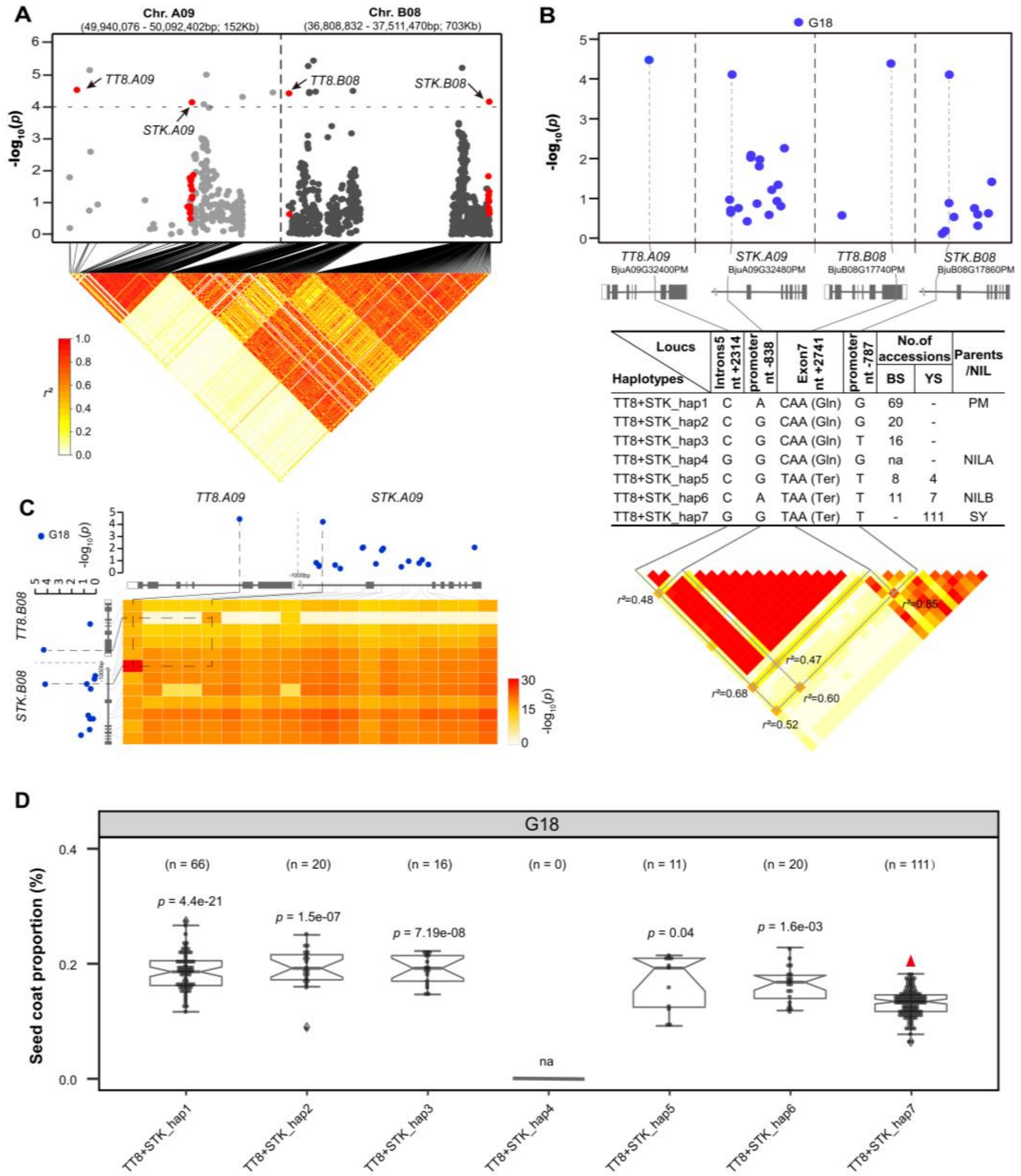

Fig. S13. Association analysis of seed coat proportion on chromosomes A09 and B08. (*A*) Manhattan plot showing two haplotypes (49,940,076 - 50,092,402 bp and 36,832 - 37,511,470 bp on chromosomes A09 and B08, respectively) significantly associated with seed coat proportion. The gray dotted line indicates the significance threshold -$\log_{10}(p)$ = 4.0. The red plots represent the position of these SNPs in two homologous *TRANSPARENT TESTA 8* (*TT8*) and *SEEDSTICK* (*STK*) genes on chromosomes A09 and B08. Heatmaps showed strong linkage disequilibrium between these SNPs, particularly in the *TT8* and *STK* gene regions. (*B*) The SNP each located in intron 5 of *TT8.A09*, the promoter of *STK.A09*, exon 7 of *TT8.B08*, and the promoter of *STK.B08* exhibited significantly associated with seed coat proportion on chromosomes A09 and B08. Seven haplotypes with frequency greater than 0.01 were identified in the *TT8* and *STK* gene regions, respectively. (*C*)

These four SNP-SNP interaction pairs were detected in *TT8* and *STK* gene regions using R package SIPI 1.01. (*D*) Boxplots for ration of seed coat to endosperm based on the haplotypes (Hap.) for two homologous *TT8* and *STK* gene copies under four different environments. Box edges represent the 0.25 quantile and 0.75 quantile with the median values shown by bold lines. Whiskers extend to data no more than 1.5 times the interquartile range, and remaining data are indicated by dots. *P*-values were calculated with Student's two tailed t-test. G18: Guiyang, China.

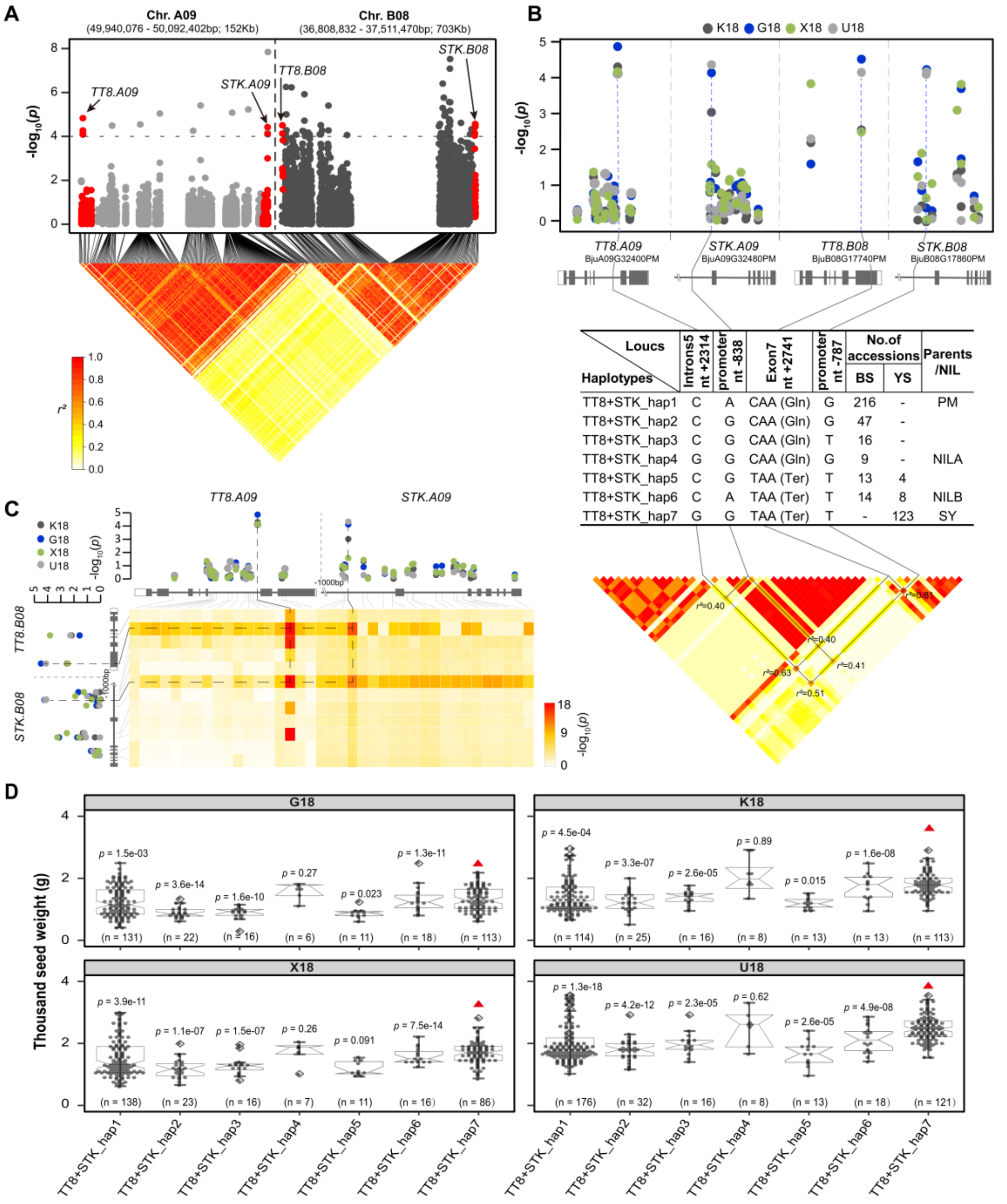

Fig. S14. Association analysis of thousand seed weight with loci on chromosome A09 and B08 of *Brassica juncea*. (*A*) Manhattan plot showing two haplotypes (49,940,076 - 50,092,402 bp and 36,808,832 - 37,511,470 bp on chromosomes A09 and B08, respectively) significantly associated with thousand seed weight. The gray dotted line indicates the significance threshold -$\log_{10}(p)$ = 4.0. The red plots represent the position of these SNPs in two homologous *TT8* and *STK* gene copies on chromosomes A09 and B08. Heatmaps showed strong linkage disequilibrium between these SNPs, particularly in the *TT8* and *STK* gene regions. Heatmaps spanning the SNP markers in linkage disequilibrium with the most strongly associated SNPs in *TRANSPARENT TESTA 8* (*TT8*) and

SEEDSTICK (*STK*) gene region. (*B*) The SNP each located in intron 5 of *TT8.A09*, the promoter of *STK.A09*, exon 7 of *TT8.B08*, and the promoter of *STK.B08* exhibited significantly associated with thousand seed weight on chromosomes A09 and B08. Seven haplotypes with frequency greater than 0.01 were identified in the *TT8* and *STK* gene regions, respectively. (*C*) These four SNP-SNP interaction pairs were detected in *TT8* and *STK* gene regions using R package SIPI 1.01. (*D*) Boxplots for thousand seed weight based on the haplotypes for two homologous *TT8* and *STK* under four different environments, respectively. Box edges represent the 0.25 quantile and 0.75 quantile with the median values shown by bold lines. Whiskers extend to data no more than 1.5 times the interquartile range, and remaining data are indicated by dots. G18: Guiyang, China, 2018. X18: Xiangtan, Hunan, China, 2018. K18: Kunming, China, 2018. U18: Urumqi, China, 2018. *P*-values were calculated with Student's two tailed t-test.

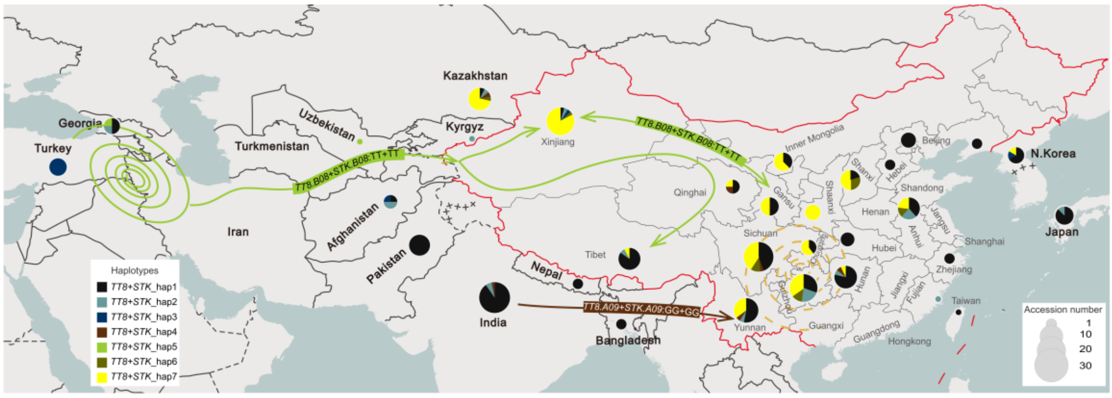

Fig. S15. Co-evolution of *SEEDSTICK* (*STK*) with *TRANSPARENT TESTA 8* (*TT8*) genes in *Brassica juncea*. A total of seven haplotypes combining *TT8s* and *STKs* were detected in *B. juncea*.

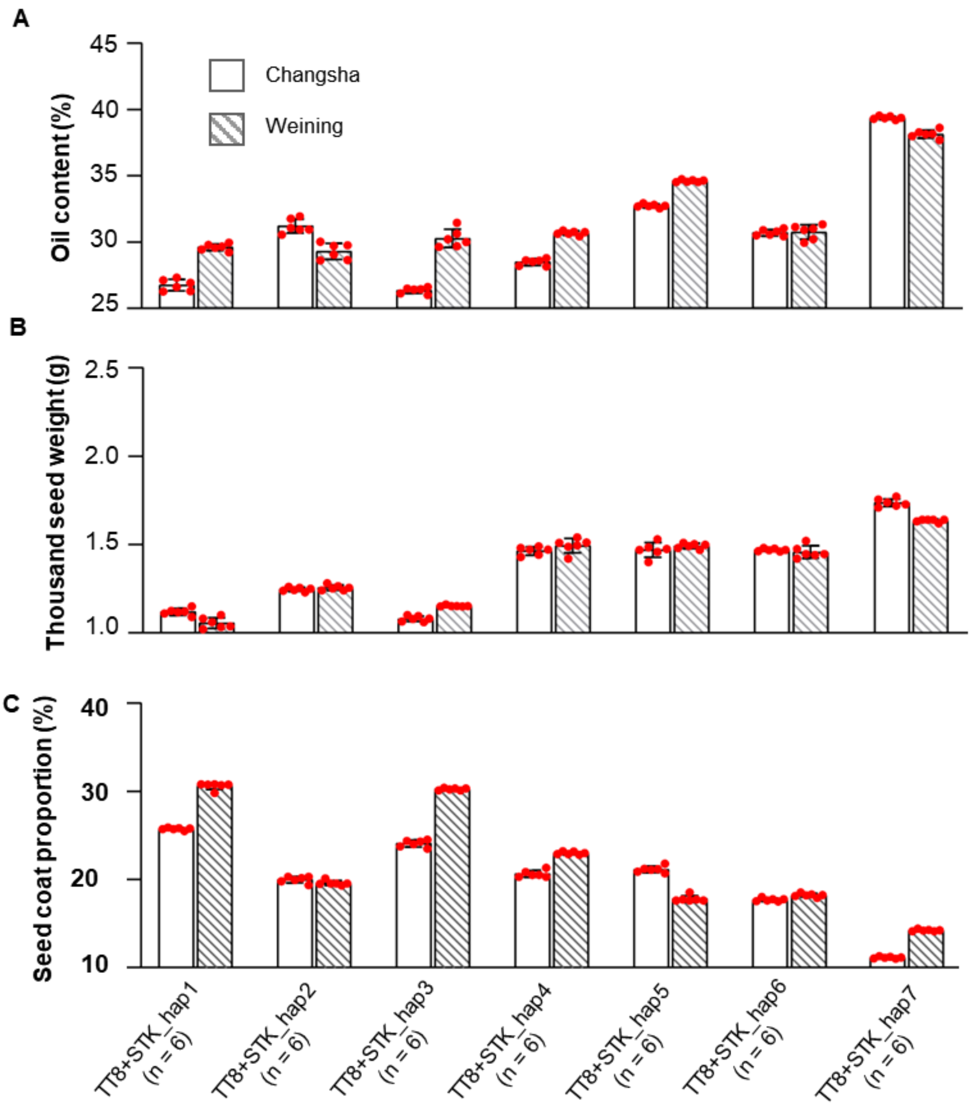

Fig. S16. Seven haplotypes corresponding to seed oil content, thousand seed weight, and seed coat proportion in Changsha and Weining. (*A*) Seed oil content; (*B*) Thousand seed weight; (*C*) Seed coat proportion. Hap: haplotype.

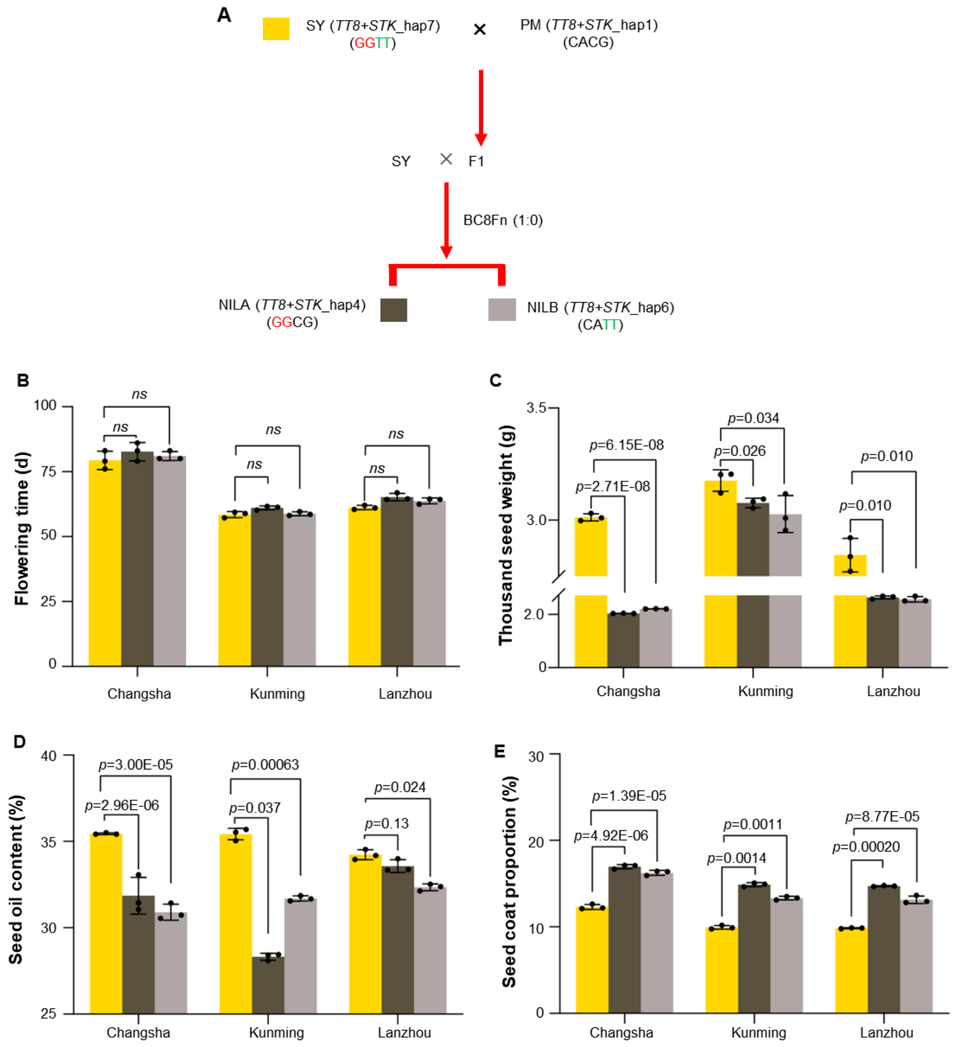

Fig. S17. Comparative analysis of six traits phenotypes between a yellow-seeded Sichuan Yellow (SY), and near isogenic line A and B (NILA and NILB) accessions of *Brassica juncea* in Changsha, Kunming and Lanzhou. (*A*) Flowering time; (*B*) Seed coat proportion; (*C*) Seed oil content; (*D*) Thousand seed weight.

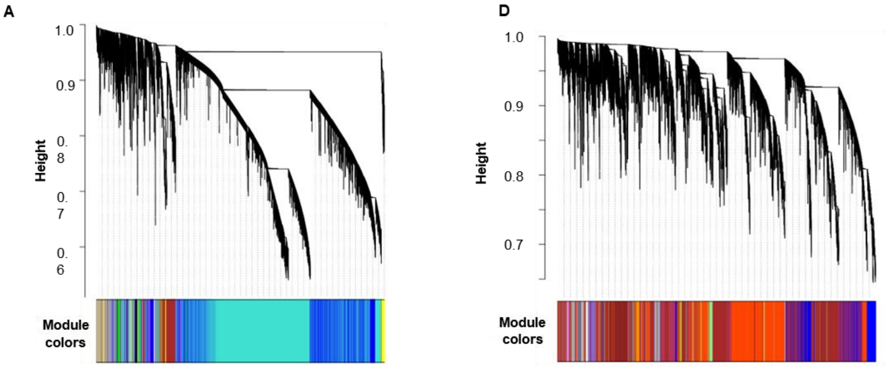

B

Module color		Module Trait coefficient	Module gene numbers
lightyellow		−0.14	96
grey60		−0.00	114
green		−0.17	987
red		0.14	588
black		−0.09	487
blue		0.47***	5595
turquoise		0.44***	12334
orange		−0.16	46
lightgreen		−0.16	104
pink		−0.20	350
darkred		−0.18	79
royalblue		0.24	81
cyan		0.30*	158
greenyellow		−0.09	215
lightcyan		0.23	118
midnightblue		0.37**	150
darkturquoise		0.45***	57
purple		0.27*	236
darkgreen		−0.29*	78
tan		0.15	212
brown		0.20	1232
yellow		−0.09	941
darkgrey		0.12	52
magenta		0.35**	334
salmon		0.34**	164
grey		0.40**	3421

E

Module color		Module Trait coefficient	Module gene numbers
antiquewhite2		0.02	181
bisque4		-0.10	248
blue		0.43**	7633
brown		-0.20	13894
brown4		-0.05	4163
coral1		0.39**	162
coral3		0.09	787
darkgrey		-0.19	575
darkorange		0.40**	693
greenyellow		0.61***	1104
indianred3		-0.17	257
indianred4		0.34*	175
lavenderblush2		0.02	88
lightcyan1		0.38**	342
lightgreen		0.02	1203
lightpink3		0.26	93
lightsteelblue		0.31*	136
maroon		-0.46***	2174
mediumpurple3		-0.01	1087
orangered1		-0.35*	19020
sienna4		0.35*	192
skyblue1		-0.11	141
skyblue2		-0.88***	891
steelblue		0.34*	415
violet		-0.33*	379

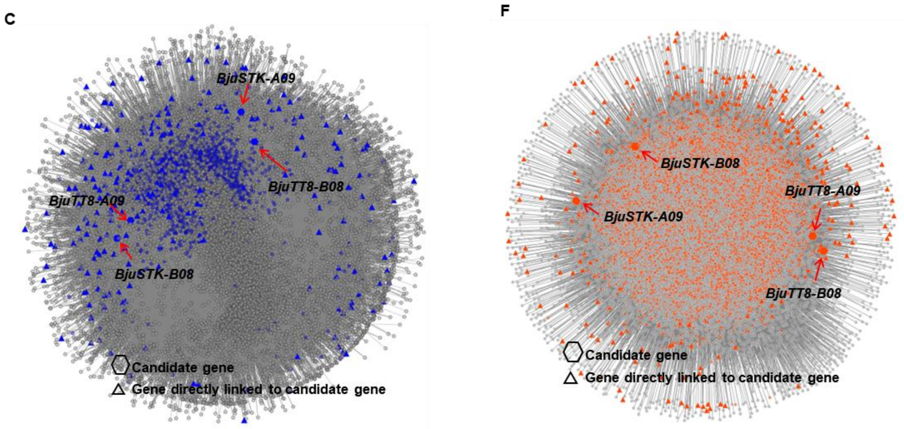

Fig. S18. Co-expression network analysis of genes at different developmental stages of the seed coat and seed in *Brassica juncea*. The clustering dendrogram identifying the weighted gene co-expression network analysis (WGCNA) modules of seed coat (*A*) and seed (*D*), respectively. Identified modules gene number and correlation with the seed color from seed coat (*B*) and seed (*E*), respectively. Whole co-expression network visualization of seed coat (*C*) and seed (*F*), respectively. The blue hexagons represent candidate genes, and the blue tangle genes are directly linked to candidate genes. The blue color represents blue module genes. *p<0.05, **p<0.01, ***p<0.001.

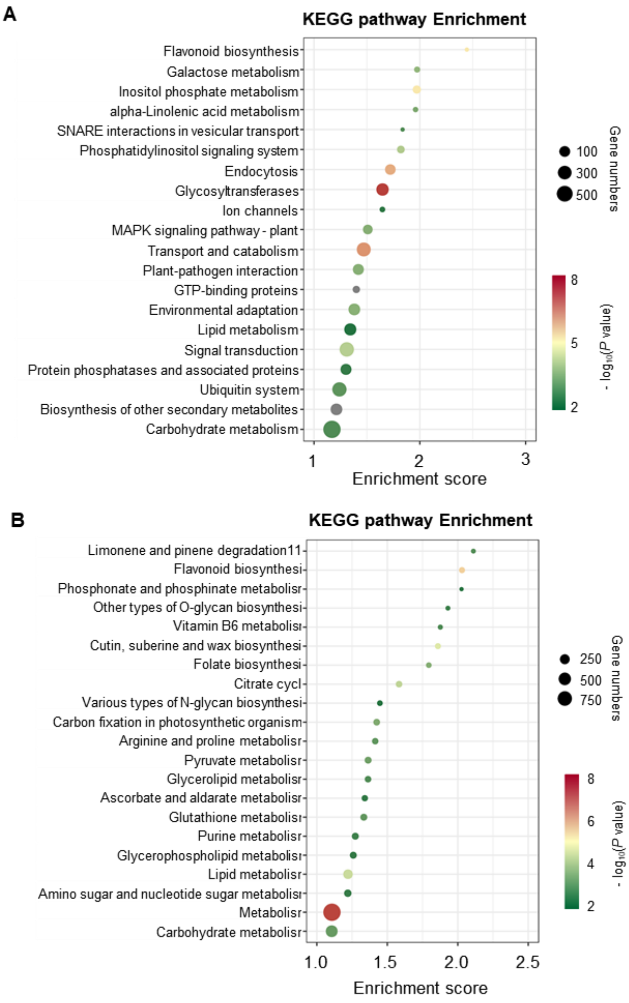

Fig. S19. Results of gene ontology (GO) enrichment and kyoto encyclopedia of genes and genomes (KEGG) analysis of genes found to be in the same co-expression module as the *TT8* genes in *B. juncea*. Significant biological process of GO enrichment in seed coat (*A*) and seed (*B*), respectively. KEGG metabolic pathway analysis in seed coat (*C*) and seed (*D*), respectively.

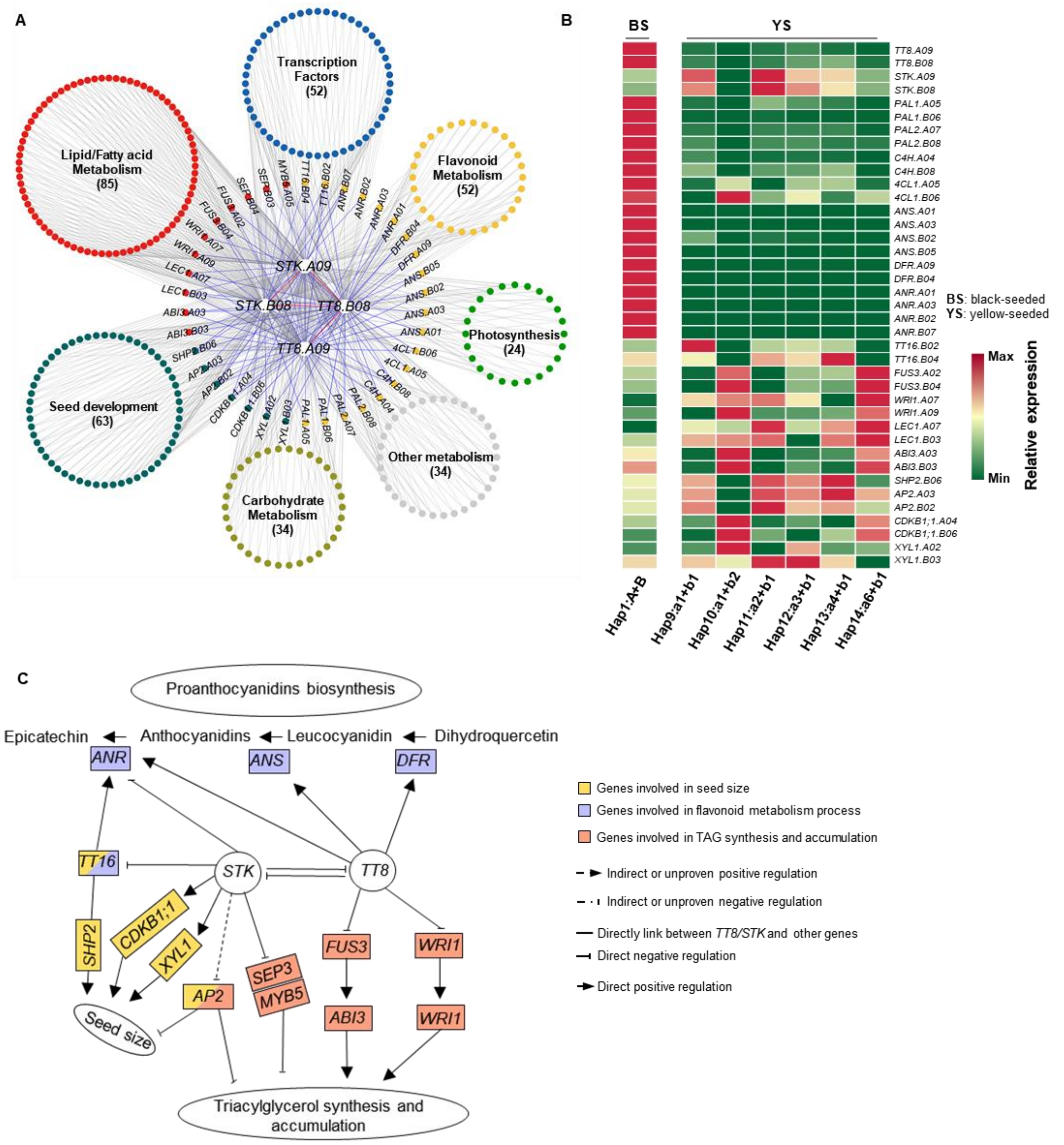

Fig. S20. The molecular regulatory network of *SEEDSTICK* (*STK*) with *TRANSPARENT TESTA 8* (*TT8*) genes involved in seed. (*A*) Co-expression network of *TT8* and *STK* genes. Based on the functional annotation, co-expression networks were classified into the following groups: lipid/fatty acid metabolism (red nodes), seed development (dark cyan nodes), flavonoid metabolism (gold nodes), transcription factors (blue nodes), carbohydrate metabolic (khaki nodes) and photosynthesis (green nodes). Genes in each category in close linkage with *TT8* and *STK* genes are indicated. (*B*) Expression analysis of flavonoid, oil content and seed weight genes in the seed coat 15 days after pollination in the wild type (Hap1) and six different mutants (Hap9 - Hap14). (*C*) A proposed model for the function of *TT8* and *STK* genes in the regulation of seed coat, seed weight and oil content. Hap: haplotype.

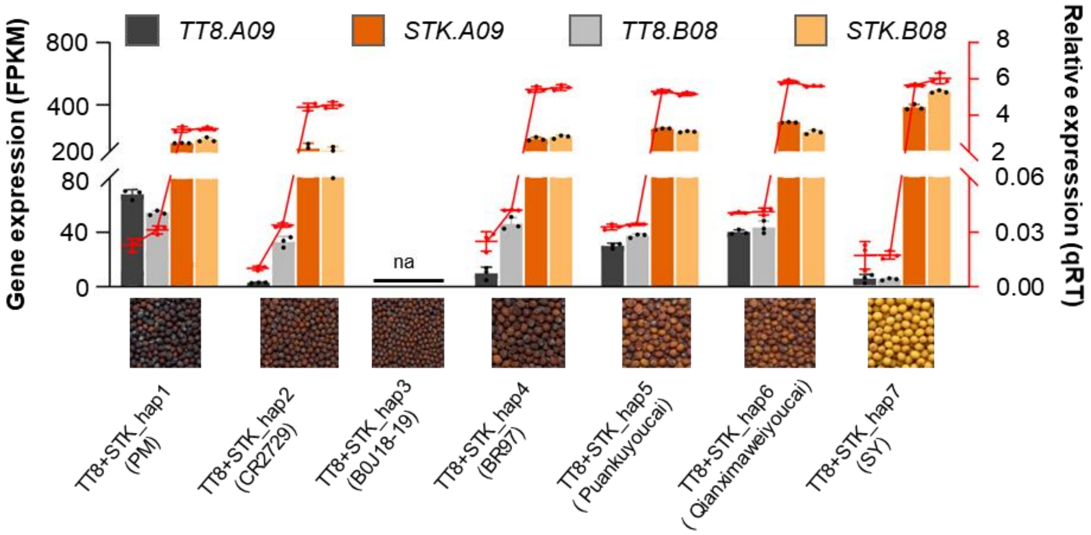

Fig. S21. Expression analysis of *SEEDSTICK* (*STK*) with *TRANSPARENT TESTA 8* (*TT8*) genes in seven haplotypes by (FPKM) and qRT-PCR. FPKM: Fragments per kilo base per million mapped. qRT-PCR: Quantitative real-time PCR.

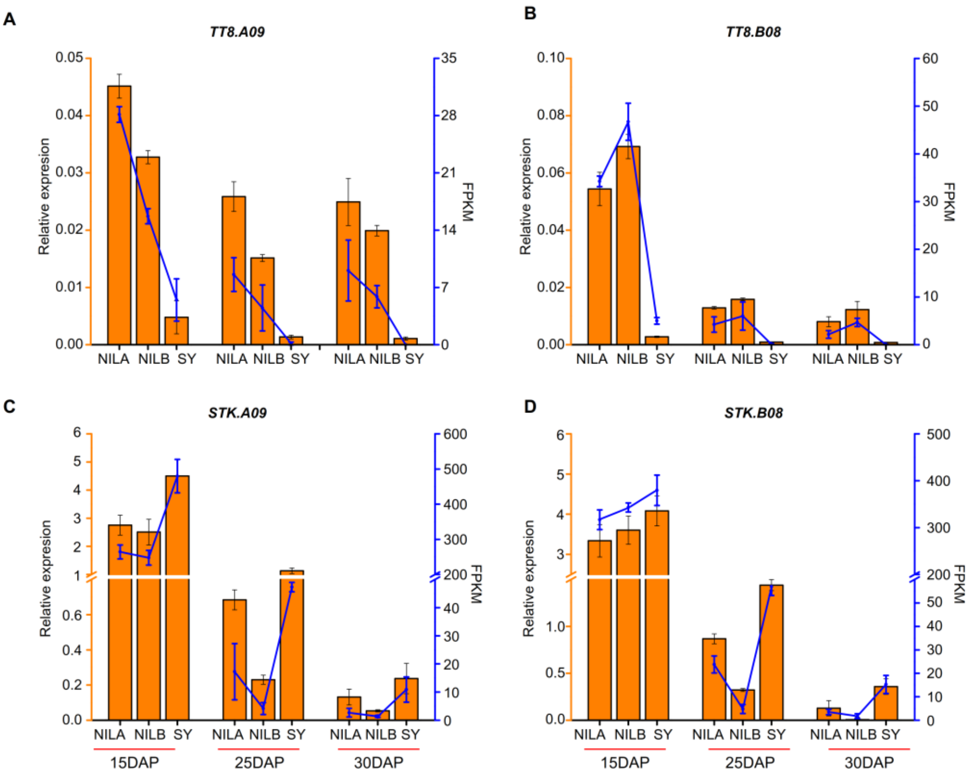

Fig. S22. Expression analysis of *TRANSPARENT TESTA 8* (*TT8*) (*A* and *B*) and *SEEDSTICK* (*STK*) (*C* and *D*) in the seed coat 15, 25, and 35 days after pollination between the NILA/B and Sichuan Yellow (SY) accessions by FPKM and qRT-PCR. FPKM: Fragments per kilo base per million mapped. FPKM: Fragments per kilo base per million mapped. qRT-PCR: Quantitative real-time PCR. DAP: days after pollination.

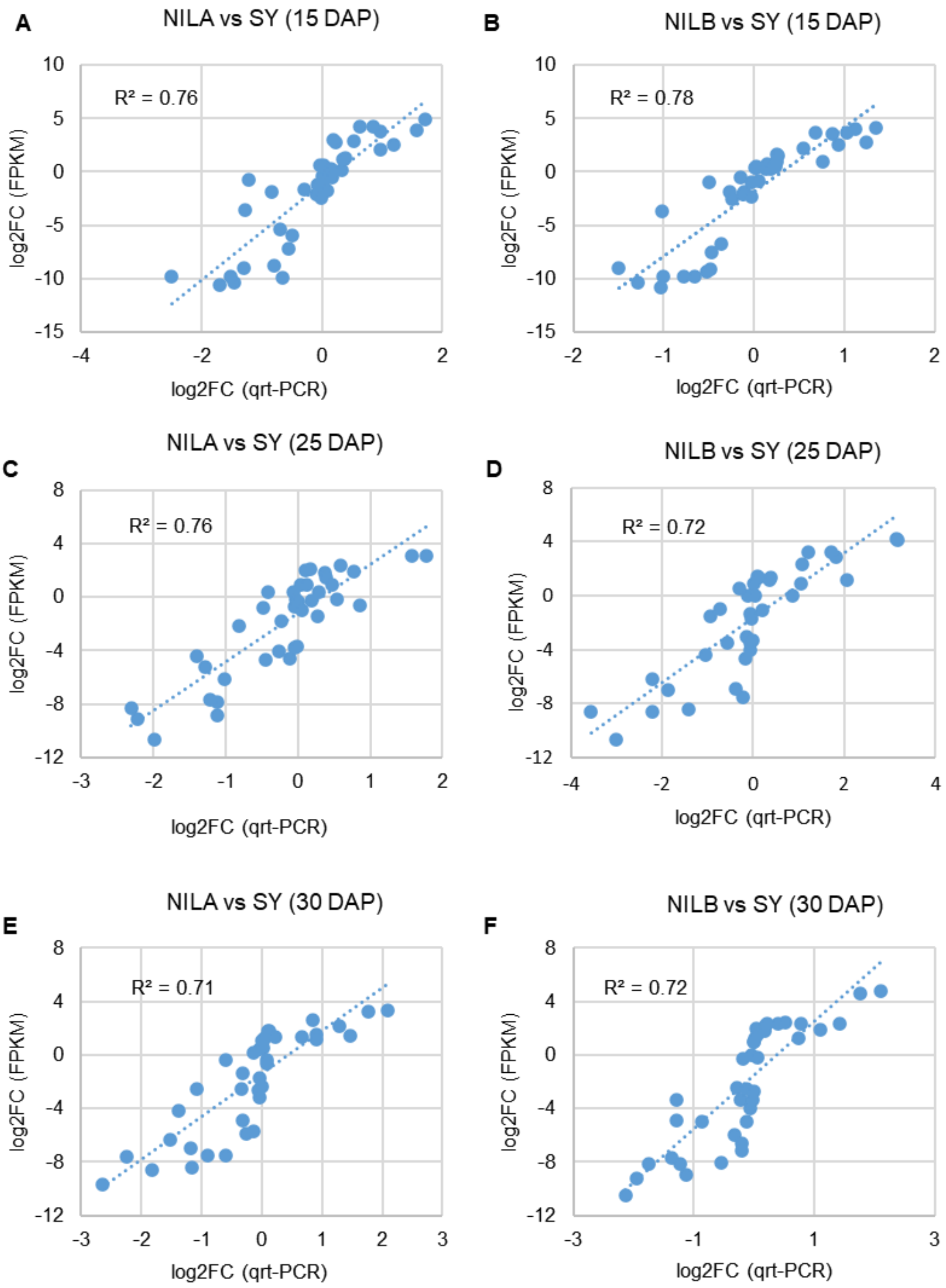

Fig. S23. qRT-PCR validation of 42 differentially expressed genes (DEGs) from Fig. 6b in seed coat. Correlation between qRT-PCR and RNA-seq for select 35 DEGs in the seed coat 15 (*A* and *B*), 25 (*C* and *D*), and 35 (*E* and *F*) days after pollination in near isogenic line A/B (NILA/B) and Sichuan Yellow (SY) is also shown. qRT-PCR: Quantitative real-time PCR. FPKM: Fragments per kilo base per million mapped. DAP: days after pollination.

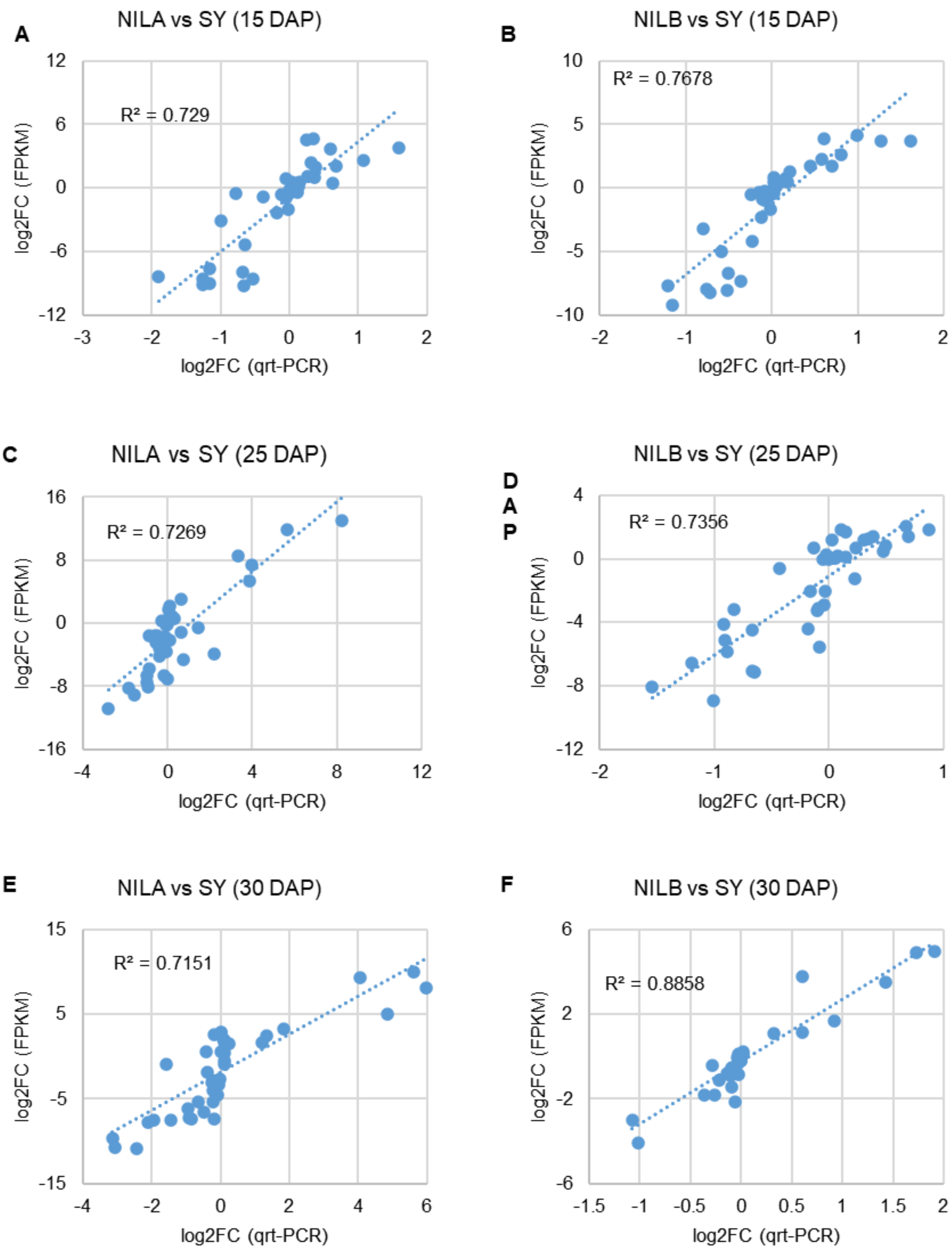

Fig. S24. qRT-PCR validation of 42 differentially expressed genes (DEGs) from Supplementary Fig. 20b in seed. Correlation between qRT-PCR and RNA-seq for select 35 DEGs in the seed 15 (*A* and *B*), 25 (*C* and *D*), and 35 (*E* and *F*) days after pollination in near isogenic line A/B (NILA/B) and Sichuan Yellow (SY) is also shown. FPKM: Fragments per kilo base per million mapped. qRT-PCR: Quantitative real-time PCR. DAP: days after pollination.

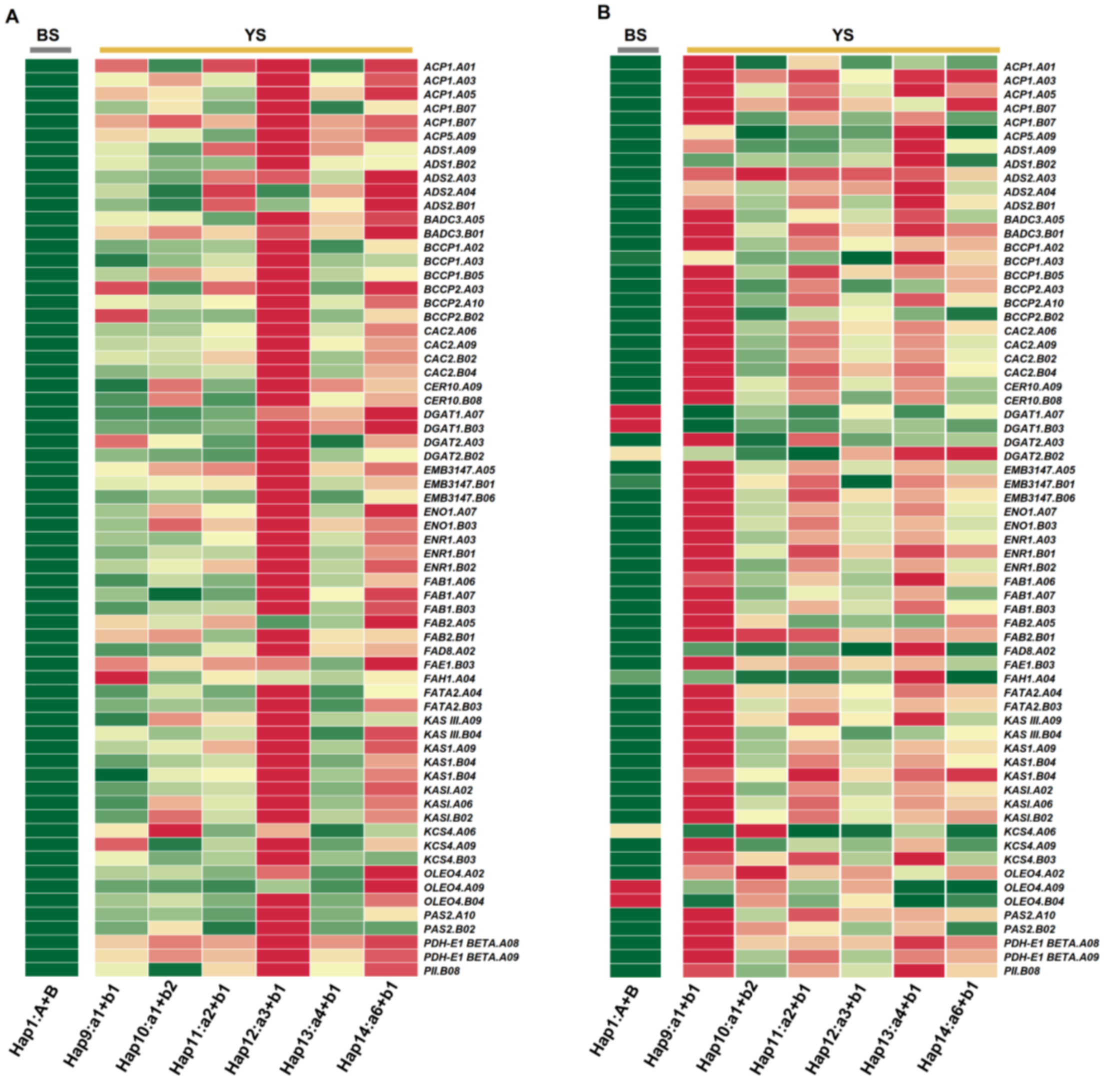

Fig. S25. The expression analysis of lipid/fatty acid synthesis genes between black- (Hap 1) and yellow-seeded (Hap 9 – Hap 14) in the seed coat (*A*) and seed (*B*) of *B. juncea.* Hap: Haplotype.

Reference

1. K. B. Lim et al., Characterization of rDNAs and tandem repeats in the heterochromatin of *Brassica rapa*. *Mol. Cells* **19**, 436–444 (2005).
2. K. B. Lim et al., Characterization of the centromere and peri-centromere retrotransposons in *Brassica rapa* and their distribution in related *Brassica* species. *Plant J.* **49**, 173–183 (2007).
3. S. Perumal et al., A high-contiguity *Brassica nigra* genome localizes active centromeres and defines the ancestral *Brassica* genome. *Nat. Plants* **6**, 929–941 (2020).
4. C. J. Schelfhout et al., A PCR-based B-genome-specific marker in *Brassica* species. *Theor. Appl. Genet.* **109**, 917–921 (2004).
5. G. Wang et al., ChIP-cloning analysis uncovers centromere-specific retrotransposons in *Brassica nigra* and reveals their rapid diversification in *Brassica* allotetraploids. *Chromosoma* **128**, 119–131 (2019).
6. C. Camacho et al., BLAST+: architecture and applications. *BMC Bioinform*. **10**, 421 (2009).
7. H. Li, Minimap2: pairwise alignment for nucleotide sequences. *Bioinform*. **34**, 3094–3100 (2018).
8. H. Li et al., The sequence alignment/map format and SAMtools. *Bioinform*. **25**, 2078–2079 (2009).
9. F. A. Simão et al., BUSCO: Assessing genome assembly and annotation completeness with single-copy orthologs. *Bioinform.* **31**, 3210–3212 (2015).
10. S. Ou et al., Assessing genome assembly quality using the LTR Assembly Index (LAI). *Nucleic Acids Res*. **46**, e126 (2018).
11. Z. Xu, H. Wang, LTR-FINDER: An efficient tool for the prediction of full-length LTR retrotransposons. *Nucleic Acids Res*. **35**, W265–W268 (2007).
12. E. Birney et al., GeneWise and genomewise. *Genome Res*. **14**, 988–95 (2004).
13. M. Stanke et al., Using native and syntenically mapped cDNA alignments to improve de novo gene finding. *Bioinform*. **24**, 637–644 (2008).
14. I. Korf, Gene finding in novel genomes. *BMC Bioinform*. **5**, 59 (2004).
15. W. H. Majoros, M. Pertea, S. L. Salzberg, TigrScan and GlimmerHMM: two open-source ab initio eukaryotic gene-finders. *Bioinform*. **20**, 2878–2879 (2004).
16. R. Guigo, Assembling genes from predicted exons in linear time with dynamic programming. *J. Comput. Biol*. **5**, 681–702 (1998).

17. C. Burge, S. Karlin, Prediction of complete gene structures in human genomic DNA. *J. Mol. Biol.* **268**, 78–94 (1997).
18. M. G. Grabherr et al., Full-length transcriptome assembly from RNA-Seq data without a reference genome. *Nat. Biotechnol.* **29**, 644–652 (2011).
19. B. J. Haas et al., Improving the Arabidopsis genome annotation using maximal transcript alignment assemblies. *Nucleic Acids Res.* **31**, 5654–5666 (2003).
20. B. J. Haas et al., Automated eukaryotic gene structure annotation using EVidenceModeler and the program to assemble spliced alignments. *Genome Biol.* **9**, R7 (2008).
21. P. Jones et al., InterProScan 5: Genome-scale protein function classification. *Bioinform.* **30**, 1236–1240 (2014).
22. T. M. Lowe, S. R. Eddy, tRNAscan-SE: a program for improved detection of transfer RNA genes in genomic sequence. *Nucleic Acids Res.* **25**, 955–964 (1997).
23. E. P. Nawrocki, S. R. Eddy, Infernal 1.1: 100-fold faster RNA homology searches. *Bioinform.* **29**, 2933–2935 (2013).
24. L. Kang et al., Genomic insights into the origin, domestication and diversification of *Brassica juncea*. *Nat. Genet.* **53**, 1392–1402 (2021).
25. Y. Lu et al., A simple and rapid procedure for identification of seed coat colour at the early developmental stage of *Brassica juncea* and *Brassica napus* seeds. *Plant Breed.* **131**, 176–179 (2012).
26. G. A. Churchill, R.W. Doerge, Empirical threshold values for quantitative trait mapping. *Genetics* **138**, 963–971 (1994).
27. X. Liu et al., Genome-wide identification, localization, and expression analysis of proanthocyanidin-associated genes in *Brassica*. *Front. Plant Sci.* **7**, 1831 (2016).
28. L. T. Nguyen et al., IQ-TREE: a fast and effective stochastic algorithm for estimating maximum-likelihood phylogenies. *Mol. Biol. Evol.* **32**, 268–274 (2015).
29. Z. Yang, PAML 4: phylogenetic analysis by maximum likelihood. *Mol. Biol. Evol.* **24**, 1586–1591 (2007).
30. D. Kim, et al., TopHat2: accurate alignment of transcriptomes in the presence of insertions, deletions and gene fusions. *Genome Biol.* **14**, R36 (2013).
31. A. Mortazavi et al., Mapping and quantifying mammalian transcriptomes by RNA-seq. *Nat. Methods* **5**, 621–628 (2018).

32. L. Wang et al., DEGseq: an R package for identifying differentially expressed genes from RNA-seq data. *Bioinform.* **26**, 136–8 (2010).
33. H. Wickham, ggplot2: elegant graphics for data analysis. New York: Springer-Verlag New York. Retrieved from https://ggplot2.tidyverse.org. (2016).
34. R. Chandna et al., Evaluation of candidate reference genes for gene expression normalization in *Brassica juncea* using real time quantitative RT-PCR. *PLoS One* **7**, e36918 (2012).
